# Understanding Voltage Gating of *Providencia stuartii* Porins at Atomic Level

**DOI:** 10.1371/journal.pcbi.1004255

**Published:** 2015-05-08

**Authors:** Wanling Song, Harsha Bajaj, Chady Nasrallah, Hualiang Jiang, Mathias Winterhalter, Jacques-Philippe Colletier, Yechun Xu

**Affiliations:** 1 CAS Key Laboratory of Receptor Research, Drug Discovery and Design Centre, Shanghai Institute of Materia Medica, Chinese Academy of Sciences (CAS), Shanghai, China; 2 School of Engineering and Sciences, Jacobs University Bremen, Bremen, Germany; 3 University Grenoble Alpes, Institut de Biologie Structurale (IBS), Grenoble, France; 4 CNRS, Institut de Biologie Structurale (IBS), Grenoble, France; 5 CEA, Institut de Biologie Structurale (IBS), Grenoble, France; University of Virginia, UNITED STATES

## Abstract

Bacterial porins are water-filled β-barrel channels that allow translocation of solutes across the outer membrane. They feature a constriction zone, contributed by the plunging of extracellular loop 3 (L3) into the channel lumen. Porins are generally in the open state, but undergo gating in response to external voltages. To date the underlying mechanism is unclear. Here we report results from molecular dynamics simulations on the two porins of *Providenica stuartii*, Omp-Pst1 and Omp-Pst2, which display distinct voltage sensitivities. Voltage gating was observed in Omp-Pst2, where the binding of cations in-between L3 and the barrel wall results in exposing a conserved aromatic residue in the channel lumen, thereby halting ion permeation. Comparison of Omp-Pst1 and Omp-Pst2 structures and trajectories suggests that their sensitivity to voltage is encoded in the hydrogen-bonding network anchoring L3 onto the barrel wall, as we observed that it is the strength of this network that governs the probability of cations binding behind L3. That Omp-Pst2 gating is observed only when ions flow against the electrostatic potential gradient of the channel furthermore suggests a possible role for this porin in the regulation of charge distribution across the outer membrane and bacterial homeostasis.

## Author Summary

Porins are the main conduits for hydrophilic nutrients and ions uptake into the periplasm of Gram-negative bacteria. Their translocation permeability is determined by the amino-acid distribution on their extracellular loop L3. Bacterial porin channels have long been known to undergo step-wise gating, under the application of a transmembrane potential. Yet the exact molecular mechanism by which gating is achieved and the exact relevance of this evolved characteristic remain elusive. In the present study, we report on electrophysiology experiments and molecular dynamics simulations on the two general-diffusion porins of *Providencia stuartii*, Omp-Pst1 and Omp-Pst2. Our results show that gating in Omp-Pst2 occurs as the result of L3 displacement, which follows from the binding of cations in acidic niches between L3 and the barrel wall and effects in exposing the side chain of a highly conserved aromatic residue at the tip of L3 in the channel lumen. That Omp-Pst2 displays asymmetric voltage sensitivity and that the likelihood of gating is increased when cations transit from the extracellular to the intracellular side suggests voltage-gating underlies a regulatory role in bacterial homeostasis. Rational antibiotic-design strategies based on the maximization of antibiotic penetration and accumulation at their target sites, should take this role into account.

## Introduction

The outer membrane of Gram-negative bacteria is sprinkled of homotrimeric channels comprised of three 16-stranded β-barrels. They are the principal conduits for the passive penetration of hydrophilic molecules into the periplasm, and are often referred to as the general diffusion porins. Classical examples include *Escherichia coli* OmpF [1], OmpC [2] and PhoE [1]. The general diffusion porins display a conserved β-barrel architecture with eight periplasmic turns and eight extracellular loops (L1–L8). Also conserved is the presence of a constriction zone (CZ), at mid height of the channel. The CZ is contributed by the plunging of extracellular loop L3 into the channel lumen, where it adopts a helix-turn-loop fold and interacts with the barrel wall through hydrogen bonding and Van der Waals (VDW) interactions. The amino-acid distributions in L3 and on the barrel wall opposite to it (“anti-L3 region”) determine the sieving properties of the porins, *i*.*e*. their ion specificity and size exclusion limit [3, 4]. The L3 and anti-L3 region generally display opposed charge distribution, with L3 being negatively charged and anti-L3 positively charged.

The general diffusion porins can switch from their open state to gated states when a transmembrane potential is applied [5–10], a phenomenon termed as voltage gating (VG). VG is characterized by step-wise, long-lasting closed states that persist until the transmembrane potential is suppressed. The critical voltage required for voltage gating (Vc) varies among porins, but is generally in the order of hundreds of mV. Vc can be influenced by a variety of environmental cues, including pH and salt concentration [11, 12], membrane constitution [8, 13], polarity of the transmembrane potential [9], or the presence of effectors such as oligosaccharides [13] and polyamines [14]. Accumulated evidences have suggested that voltage sensing in general diffusion porins occurs at the CZ [15–21]. It was shown that replacement of L3 charged residues by uncharged ones invariably results in alteration of voltage sensitivity, channel conductance and/or ion selectivity [15, 21, 22]. In particular, replacement of negatively charged residues from L3 leads to an increase of Vc in cation-selective *E*. *coli* OmpF, while that of the positively charged residues in anti-L3 regions causes the decrease of Vc in anion-selective *E*. *coli* PhoE [15]. In addition, mutagenesis studies on OmpF showed that destabilization of L3 by deletion of residues at its tip leads to increased voltage sensitivity (lower Vc) and reduced conductance [18, 19], whereas mutations of residues involved in L3 stabilization result in reduced voltage sensitivity (higher Vc) [19, 20].

More than a decade ago, Tieleman *et al* reported the first molecular dynamics (MD) simulation of *E*. *coli* OmpF embedded in explicit lipids. Their results revealed instability in L3 due to a breakdown of the hydrogen-bonding network (HBN) anchoring L3 to the barrel wall [23]. Suspecting that the fluctuation in the CZ may have been due to the protonation setting, Im *et al* and Varma *et al* respectively implemented MD simulations using different ionization states to the charged residues on L3 [24–26]. The invariable observation was that L3 is prone to large fluctuations, suggesting that this loop could intervene in translocation across porin and possibly also in voltage-gating. Owing to constant improvements in MD simulation algorithms [27, 28] and the successful implementation of artificial transmembrane potentials [29], it has become possible to simulate ion mobilizing within the channel and thus to study the channel transport properties at a molecular level. For example, Pezeshki *et al* showed that mutation of one charged residue within CZ leads to visible effects on ion permeation and selectivity in OmpF [4]; Faraudo *et al* found that removal of negative charges in CZ influences the distribution of cations along OmpF channel [30]. However, owing both to limited computational resources and to the low voltage sensitivity (high Vc) of the hitherto simulated porin (OmpF), the molecular mechanism by which the general diffusion porin gating occurs has not yet been reported.

In the following, we report on extensive comparison of sub-microsecond scale MD simulations that provide insights into the molecular basis of voltage gating in general diffusion porins. Simulations were conducted on Omp-Pst1 and Omp-Pst2, two general diffusion porins from *Providencia stuartii* [31]. The sequence identities with OmpF of Omp-Pst1 and Omp-Pst2 are 50% and 46.1% respectively, while the RMS deviation of their Cα atoms (as measured from their respective X-ray structures) are 0.94 Å and 0.89 Å respectively. Omp-Pst1 and Omp-Pst2 show a high level of structural similarity, but owing to a completely different pattern of charge distribution along their channel wall, the two porins display opposite ion selectivities. Furthermore, whereas Omp-Pst1 gates at voltages above 199 mV, Omp-Pst2 undergoes the typical three-step gating at voltages as low as ~20 mV, making it the most voltage sensitive bacterial porin studied to date. Taking advantage of this striking contrast, we constructed parallel simulations between Omp-Pst1 and Omp-Pst2 at positive, negative and none transmembrane potentials (V_TM_; extracellular to intracellular). In Omp-Pst2, we observed gating at V_TM_ < 0 V, but not at V_TM_ > 0 V, consistent with asymmetrical gating observed experimentally. At V_TM_ < 0 V, the gating stems from stable binding of cations in acidic niches behind L3, which in turn disrupts the HBN anchoring L3 to the barrel wall and thereby allows a local conformational change in conserved W111 at the tip of L3. The repositioning of W111 aromatic side chain in the middle of the CZ effectively halts ionic permeation across the Omp-Pst2 channel. At V_TM_ > 0 V, the HBN between L3 and the barrel wall strengthens. The stabilized L3 impedes cation binding, and thus thwarts gating. In Omp-Pst1, gating was not observed, regardless of the V_TM_ applied. Additional hydrogen bonds in HBN contribute to a more resilient L3. Altogether, our results suggest that the voltage sensitivity of Omp-Pst1 and Omp-Pst2 is encoded in the HBN that anchors L3 onto the channel wall, and that conformational changes in the side chain of conserved W111 at tip of L3 leads to channel closing.

## Results

### Single channel electrophysiology

Reconstitution of a single Omp-Pst1 / Omp-Pst2 channel in a planar lipid bilayer showed a single trimer conductance of 2.7 ± 0.1 / 3.7 ± 0.2 nS respectively at 1M KCl, pH 7. In the ion selectivity measurements performed as described in [32], Omp-Pst2 shows strong cation selectivity whereas Omp-Pst1 shows indistinctive cation selectivity (Table 1). For Omp-Pst1, the critical voltage (Vc) for observing the typical three-step gating was ≥199 mV in all measurements (n = 8). For Omp-Pst2, pore-to-pore variation was observed, and Vc measurements varied between 20 and 90 mV, with a median at 50 mV (n = 20).

**Table 1:** Conductance, ion selectivity and critical voltage of Omp-Pst1 and Omp-Pst2 measured in single channel.

Porin	Conductance* (nS)	P_K_^+^/P_Cl_^-^ **	Critical voltage (mV)
Omp-Pst1	2.7 ± 0.2	1.3	≥199
Omp-Pst2	3.7 ± 0.1	8.3 ± 1.5	20–90

* Conductance of porins was measured at 1M KCl, 10 mM HEPES at pH 7.

** Selectivity of porins (cations over anions) was measured in presence of 100 mM KCl on *cis* side and 1M KCl on *trans* side at pH7.

### Simulated ion selectivity and conductance agree with experimental data

The crystallographic structures of Omp-Pst1 and Omp-Pst2 trimers were used as starting model for the simulations. Briefly, each porin was inserted in a lipid bilayer, solvated at pH 7, and the ionic concentration was adjusted to 1 M KCl. After equilibration, the two systems were subjected to either a negative (V_TM_ direction pointing from extracellular to intracellular) or a positive transmembrane potential (V_TM_ direction pointing from intracellular to extracellular) and each simulation was ran for > 500 ns. Additional simulations without a transmembrane potential were also carried out for both porins and each lasted for 100 ns. The β-barrel remains rigid in all voltage conditions, showing RMS fluctuation lower than 1 Å (S1 Fig). Larger fluctuation is observed in extracellular loops, especially in L1, L4, L5 and L6. L1 lies on the interface of the trimer and interacts with the positively charged residues at anti-L3 regions from another monomer. L4, L5 and L6 form secondary structure elements that associate to cover the extracellular entrance of the channels. Applying transmembrane potentials does increase the amplitude of loop fluctuation. However, according to the principal component analysis, their movements are constrained in a radius of ~5 Å, as imposed by a strong network of polar interactions between adjacent loops (S2 Fig).

**Fig 1:**
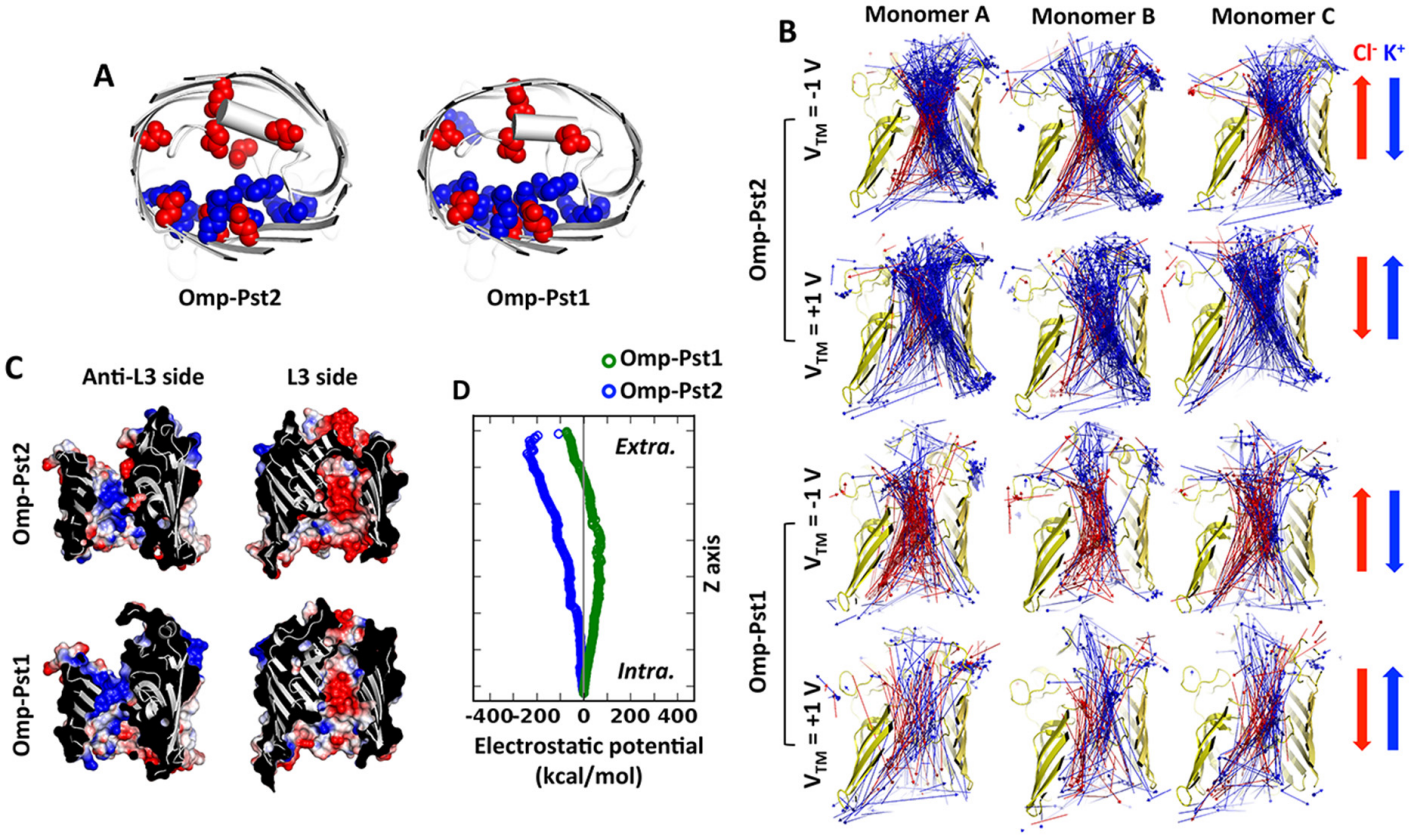
Electrostatic properties of Omp-Pst1 and Omp-Pst2. (A) Charge distribution at constriction zone. The constriction zone is defined as the area within 5 Å of the narrowest point of the channel. The negatively charged residues are shown in red spheres and the positively charged in blue spheres. (B) Mode vectors of cations (blue) and anions (red) for every 1 ns within the first 100 ns in the voltage-applied simulations. (C) Electrostatic surface of anti-L3 side and L3 side. Contour color set as ± 100 kT/e. (D) Electrostatic potentials along the pores calculated by HOLE. The values are normalized to intracellular entrance.

In simulations with V_TM_ ≠ 0, anions and cations separate into two pathways at the CZ: cations (K^+^) trail along negatively charged L3 (residues Y98 to D123 in Omp-Pst1 and Y95 to D120 in Omp-Pst2, respectively), while anions (Cl^-^) duct on the positively charged anti-L3 region (Fig 1A and 1B). In Omp-Pst1, K^+^ mainly shuffle between the acidic side chains of L3 residues D109 and D117, while Cl^-^ interact with the basic side chains of anti-L3 residues K16, R20, R41, R59, K65, R78, K163 and K170. In Omp-Pst2, these residues are D106, D114 and D117, on the one hand, and R20, R38, R56, R75, K160 and K168, on the other.

**Fig 2:**
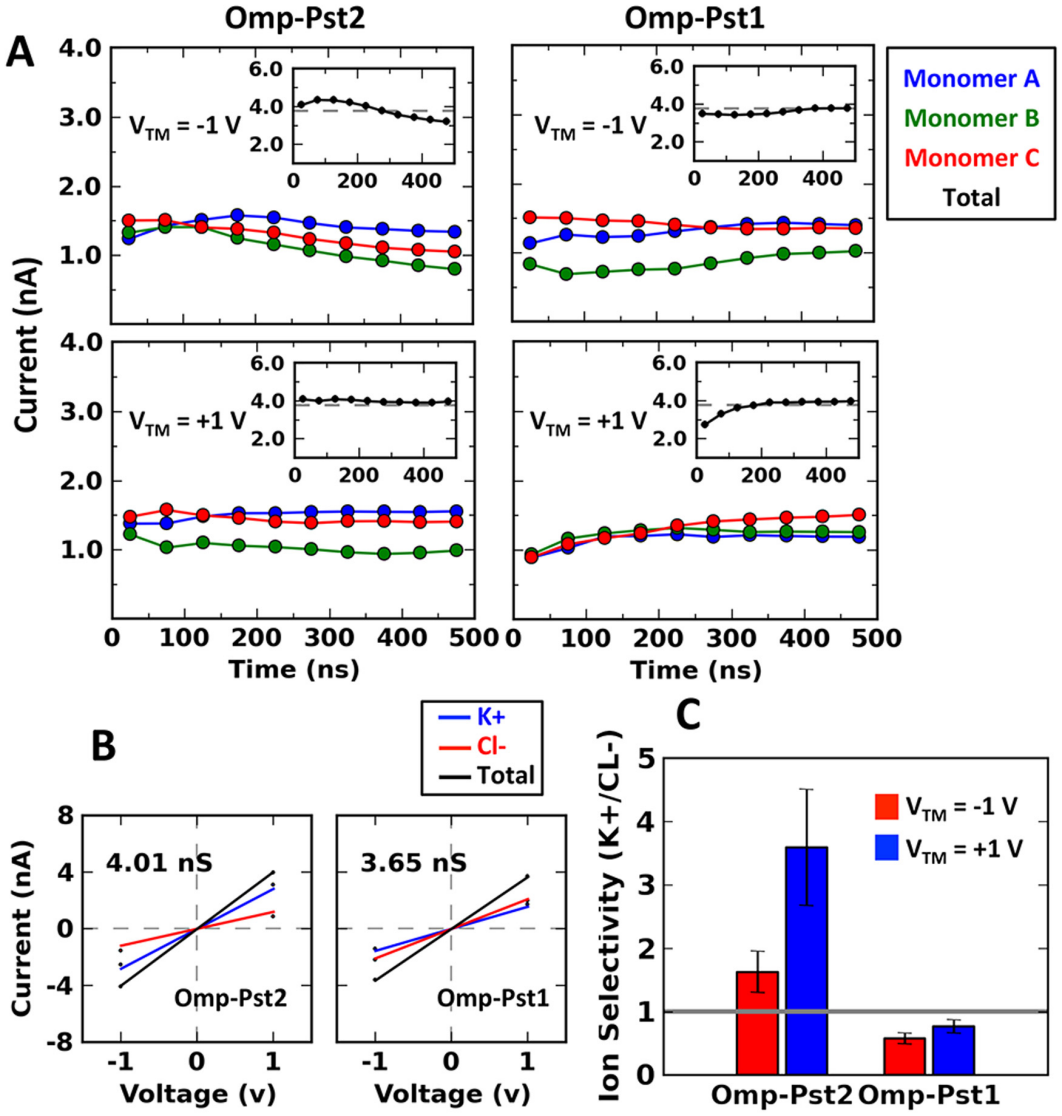
The calculated current, conductance and ion selectivity from MD simulations. (A) The currents are calculated every 50 ns monomer-wise and the total channel currents are shown in in-set panels in comparison to the experimentally determined values (dash lines). (B) The conductances of the porins’ channel are obtained from the slope of their I-V curve. The raw channel conductances are shown. (C) The ion selectivity is determined as the ratio of K^+^ to Cl^-^ current. The value shown at each voltage is the averaged ratio from three monomers and the error bar corresponds to the standard deviation among the three.

Steady ionic currents were developed in the voltage-applied systems except for Omp-Pst2 at V_TM_ < 0 mV. As we show below, gating occurred in this simulation, thus impacting ion current across the channels (Figs 2A and S3). In the other simulations, ion currents across the channel reached excellent agreement with the experimental measurements after fluctuating for ~200 ns. The current curve suggests that long equilibration time (~100–200 ns) is required when simulating porins at high ionic concentration (1M in our case). That this requirement was not fulfilled in earlier simulations on porins at high ionic concentrations may explain the discrepancy between their simulated and experimentally measured currents [4, 33]. From the stable K^+^ and Cl^-^ permeation after 200 ns or before gating in the case of Omp-Pst2 at V_TM_ < 0 mV, we derived the simulated conductance via least-square linear regression of the I/V curve (V_TM_ = [–1, 0, 1] V). The raw conductance for Omp-Pst1 and Omp-Pst2 are 3.64 nS and 4.01 nS respectively, while the corrected values based on ion diffusion coefficients (See Methods) are 3.95 nS and 4.47 nS respectively. The deviation from experimental observables might result from poor data samples in regression fitting.

Simulations indicate a strong cation-selectivity for Omp-Pst2 and a mild anion-selectivity for Omp-Pst1 (Fig 2C), in line with experimental electrophysiology data and also with the analysis of their structures. The net charge of Omp-Pst1 is indeed +1 e at the constriction zone (within 5 Å radius of channel’s narrowest point) and +5 e along the pore, while that of Omp-Pst2 is -4 e at the constriction zone and -3 e along the pore. In the case of Omp-Pst2, we observed that translocation of cations from the intracellular to the extracellular side is more efficient than the other way around (and conversely for Cl^-^ ions), as grounded by its two times more pronounced cation-selectivity at V_TM_ > 0 than at V_TM_ < 0 (Fig 2C). This asymmetry in ion selectivity correlates with the asymmetry in charge distribution (and thus with the gradient of electrostatic potential) along Omp-Pst2 channel, which features more acidic residues on the extracellular side than on the intracellular side. In Omp-Pst1, where the distribution of charged residues is even along the pore (Fig 1C), ion selectivity and translocation rate are less affected by the direction of the transmembrane potential (Fig 2C). Our simulations thus suggest that the ion selectivity of porins is not only determined by the charge distribution at their constriction zone [30, 34, 35], but also by the profile of charge distribution along the channel.

### Voltage gating in Omp-Pst2 at V_TM_ < 0 V

**Fig 3:**
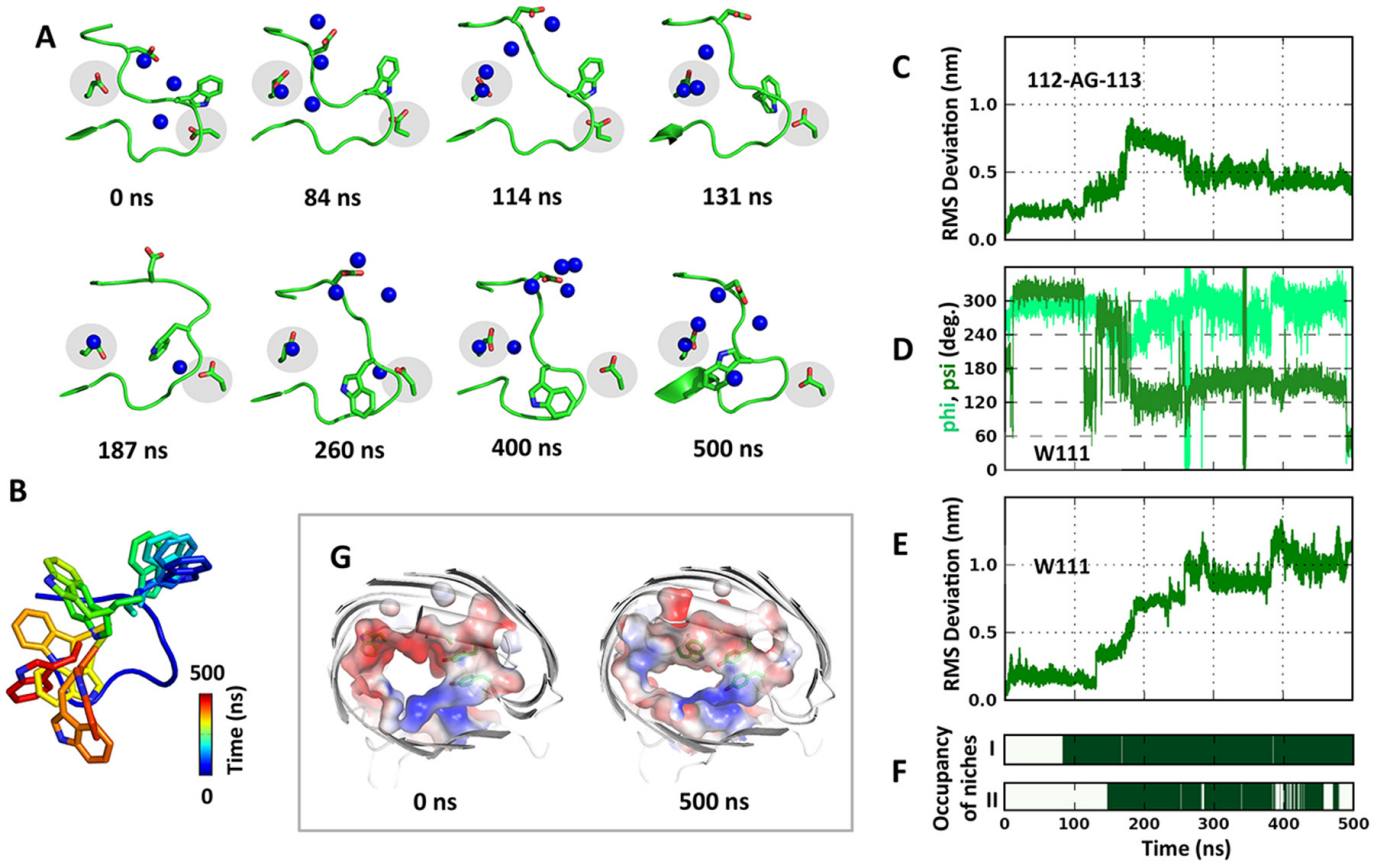
Gating process in Omp-Pst2 monomer B at negative voltage. (A) Snapshots of L3 tip. In each snapshot, residues W104 to D117 are shown in cartoon; W111, D114, E258 and D312 in sticks; and K^+^ within 3.5 Å of the L3 tip in blue spheres. The acidic niche I (E258) and niche II (D312) are highlighted in gray. (B) W111 movements in every 50 ns. The RMSD of 112-AG-113 (C), the phi/psi angle of W111 (D) and the RMSD of W111 (E) are laid out to demonstrate the sequential events leading to niches exposure and subsequent binding of cations within them (F). G) The electrostatic potential surface at 0 ns and 500 ns. The extent of color scale is ± 100 kT/e. Y95, Y99, A103 and W111 are shown in sticks.

After 100 ns, a decline in ion fluxes was observed in the simulation of Omp-Pst2 at V_TM_ < 0. Examination of ion fluxes on a monomer basis reveals that the decline mainly stems from one monomer in the trimer (monomer B) undergoing gating. At V_TM_ < 0, K^+^ ions are forced to translocate from the extracellular to the intracellular side, and inversely for Cl^-^ ions. This direction of transit is unfavourable for cations, as suggested by the electrostatic potential developed along Omp-Pst2 channel and established by the two times slower uptake of cations in comparison to the other direction. As this unfavourable flux gets heavier across the channel of monomer B, it effects in disorganizing the HBN within L3 (S4 Fig). A main-chain flip consequently occurs after 84 ns in 112-GA-113, resulting in the opening a highly acidic niche (niche I) between L3 and the barrel wall (Fig 3A). The acidic nature of the niche mainly results from the side chain oxygens of E258, a barrel-wall residue hitherto shielded from the bulk, but also from the side-chain oxygens of N102, T105, T115 and N276. A K^+^ ion rapidly (within 0.5 ns) lodges into niche I (Fig 3F), dragging along the side chain of D106. This results in the latter adopting a barrel-facing conformation, thereby reinforcing of the acidic nature of the niche. The change in orientation of D106 propagates to D114, whose side chain draws towards the channel lumen, leading to yet another main chain rearrangement in L3 (~114 ns) (Fig 3C). Originating in 112-GA-113, these changes rapidly transduce to 111-WGAD-114 (~131 ns) and result in W111 side chain wandering away from the barrel wall (Fig 3D and 3E). W111 movement effects in uncovering D312, a highly conserved barrel wall residue whose side chain oxygens hydrogen bond to the tip of L3 (main chain nitrogens of L110 and W111) in the crystallographic structure of Omp-Pst2. Exposure of D312 side chain generates a second acidic niche (niche II) in which another K^+^ ion binds ~10 ns latter (Fig 3F). Occupation of this niche effects in unleashing the tip of L3, and most notably, the side chain of W111 (Fig 3B). Originally constrained by Y20, K314 and V334, W111 first positions itself in the middle of L3 (~187 ns), but finally ends up in the channel lumen (~260 ns). There, it imposes steric and hydrophobic hindrance to ions translocation, notably through the formation of a hydrophobic belt by Y95, Y99 and A103 side chains (Fig 3G). In the last 100 ns, translocation of K^+^ ions is diminished by ~3/4 in monomer B, while that of Cl^-^ ions by ~1/4 (S5 Fig).

In the two other monomers of the Omp-Pst2 trimer simulated at V_TM_ < 0, a comparable sequence of events is observed, albeit incomplete and spanning a longer time scale. In monomer C, a potassium ion binds in niche I after ~110 ns, following a main chain flip in 112-GA-113 (~80 ns). Similar to that in monomer B, binding of this first K^+^ ion induces a conformational change in 111-WGAD-114 (~240 ns) that results in the detachment of W111 from the barrel wall (243 ns) and the concomitant opening of niche II. However, binding of a K^+^ ion in this niche occurs at more than 200 ns latter (~ 453 ns). Thus, D312 side chain remains in its native conformation and migration of W111 side chain toward the channel lumen does not complete (S6 Fig). Accordingly, only a slight reduction of K^+^ flux is observed, while Cl^-^ flux remains unaffected. In monomer A, binding of a K^+^ ion in niche I occurs after 327 ns, and the resulting conformational change in 112-GA-113 after 350 ns. By the end of the simulation time, however, no K^+^ ion lodges into niche II. W111 consequently stays in place, and ionic fluxes remain steady (S7 Fig). It is interesting to recall that in electrophysiology experiments, gating of the three monomers in a trimer also occurs sequentially. Thus monomers A, C and B could represent different Omp-Pst2 intermediates in the process of gating.

### Steadier attachment of L3 prevents gating in Omp-Pst2 at V_TM_ > 0 V

No voltage gating was observed in the simulation of Omp-Pst2 at V_TM_ > 0. This observation is consistent with experimentally observed asymmetrical gating behaviour [10, 36, 37]. Trajectory analysis reveals that it is the stronger stabilization of L3 at V_TM_ > 0 that impeaches gating. In short, a main chain rearrangement in 112-GA-113 leads to exposure of E258 and concomitant binding of K^+^ ions in niche I after ~340, 20 and 95 ns in monomers A, B and C respectively. In the two latter, main chain rearrangements in W111 (after 210 and 340 ns in monomers B and C, respectively) precede binding of K^+^ ions in niche II (after 245 and 351 ns in monomers B and C, respectively). Nevertheless, K^+^ binding to niche II is less stable at V_TM_ > 0 and gating is hence not observed (S8–S10 Figs).

**Fig 4:**
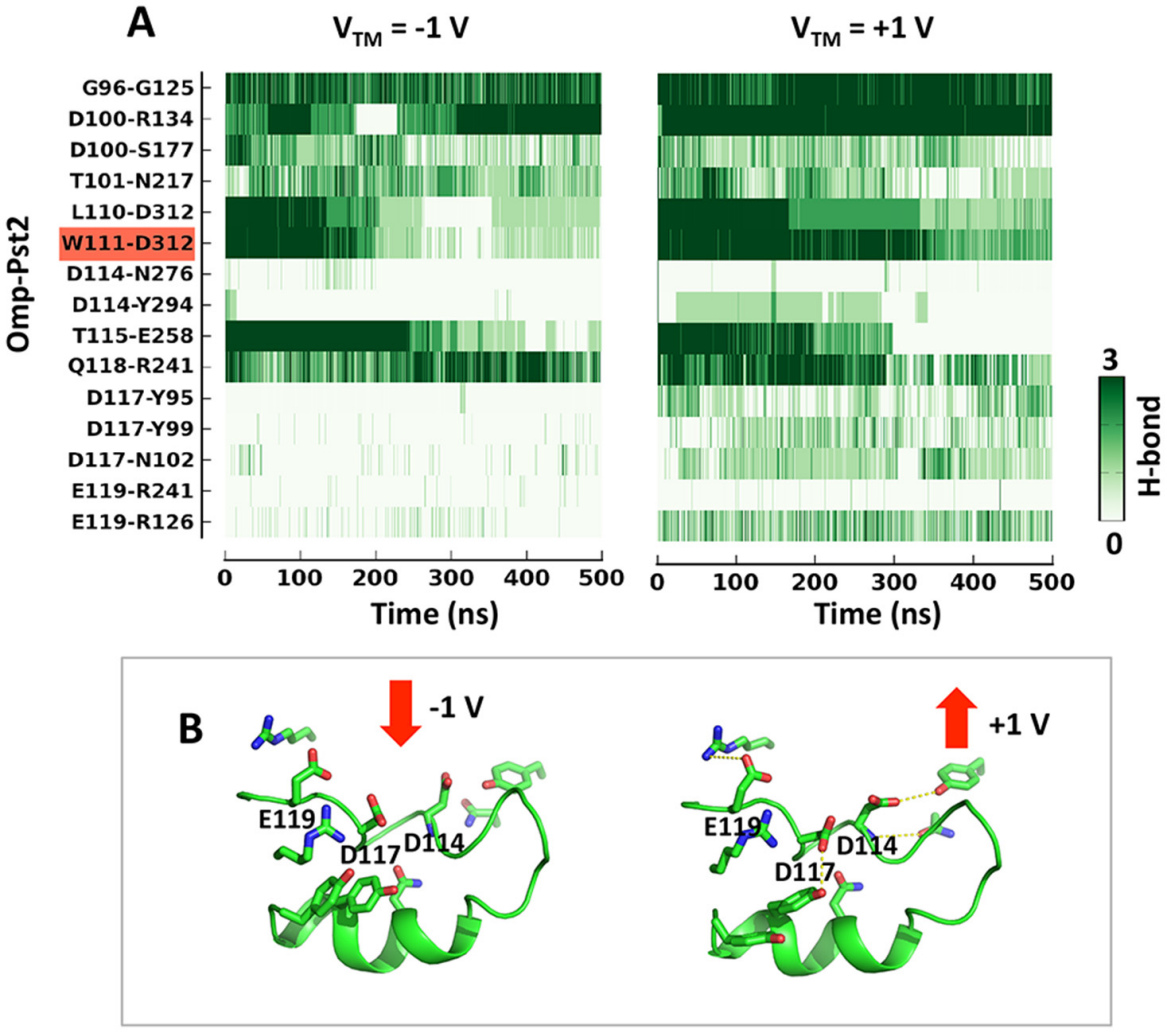
Comparison of hydrogen-bonding network on Omp-Pst2 L3 at two voltages. A) Hydrogen bonds on L3 as a function of time. The color scale indicates whether the hydrogen bond is seen in 0, 1, 2 or 3 monomers of the trimer. The hydrogen bond W111-D312, which anchors L3 tip to barrel wall, is highlighted. B) Snapshots of Omp-Pst2 L3 at 200 ns at V_TM_ = +/- 1 V. The three charged residues D114, D117, E119, and their hydrogen-bonding residues are shown in sticks.

Two factors are responsible for the reduced gating sensitivity of Omp-Pst2 at V_TM_ > 0. First and most importantly, the HBN anchoring L3 to the barrel wall is more robust at V_TM_ > 0 than at V_TM_ < 0 (Fig 4A), owing to a more favourable orientation of acidic side chains. At V_TM_ > 0, the acidic residues on Omp-Pst2 L3 are indeed facing toward the barrel wall, facilitating interactions with non-L3 residues. Major stabilizing interactions include: i/ a salt-bridge between E119 and R126; ii/ alternating hydrogen bonds between D117 and either Y99 or N102; and iii/ an hydrogen bond between D114 and Y294 before the main chain rearrangements in 112-AG-113 occurs (Fig 4B). In strong contrast, L3 acidic residues adopt outward conformations in the simulation at V_TM_ < 0, which prevents them from forming hydrogen bonds or salt-bridges with neighbouring residues. Secondly, that the transit of K+ ions is energy favoured at V_TM_ > 0 and that these ions thus transit faster across the CZ furthermore diminishes their likelihood of forming stable interaction with D312. Their intermittent binding to niche II does not trigger the repositioning of W111 side chain.

### L3 fastening prevents gating in Omp-Pst1

**Fig 5:**
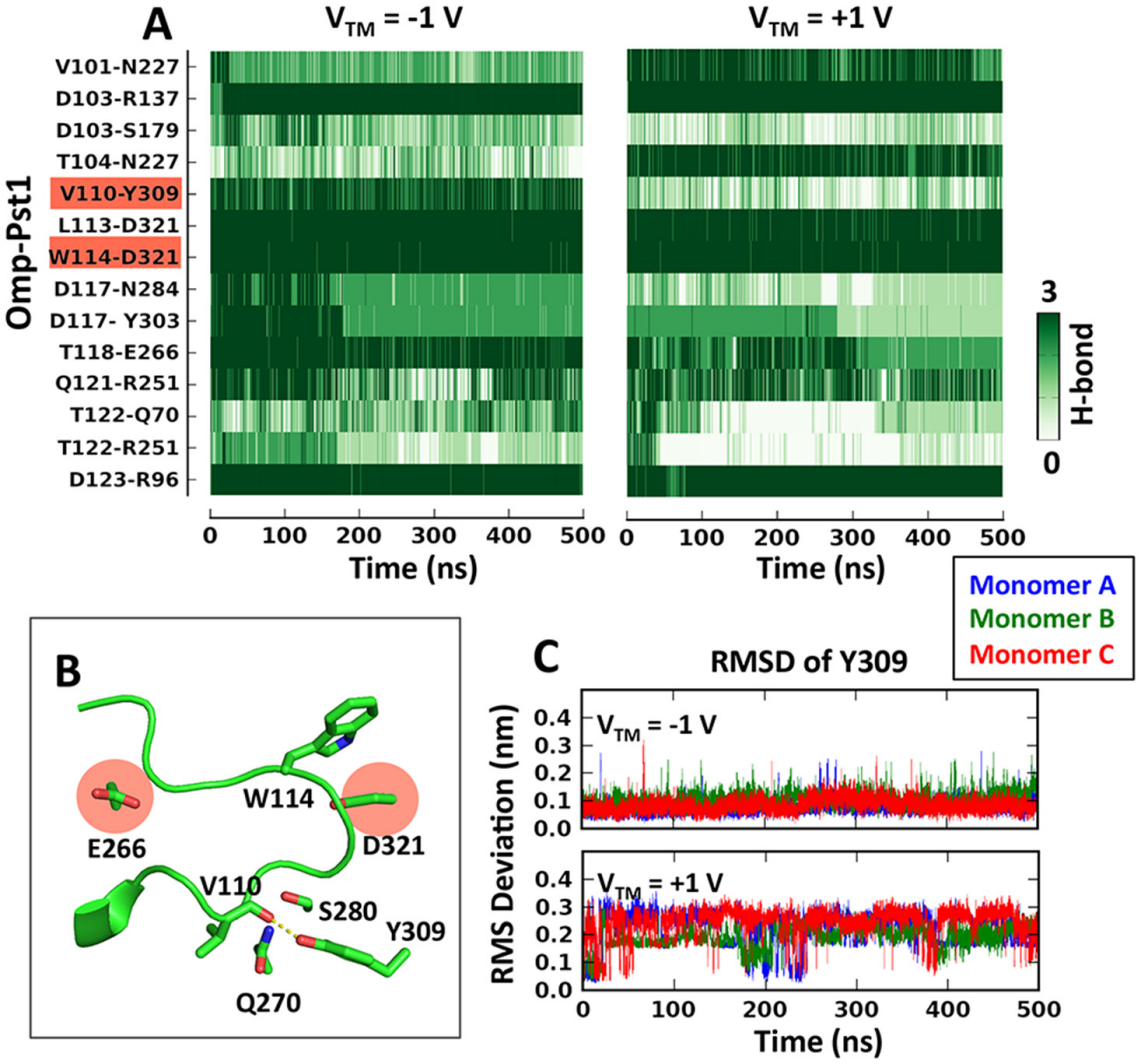
Hydrogen-bond network on Omp-Pst1 L3. (A) Hydrogen-bonds on L3 as a function of time. The color scheme is the same as in Fig 4A. Hydrogen bonds V110-Y309 and W114-D321 are highlighted. (B) Structure of Omp-Pst1 L3. The hydrogen bond between V110 and Y309 highlighted in panel A is labeled out in dash lines. (C) RMS Deviation of Y309 as a function of time in three monomers at V_TM_ = +/- 1 V.

From a structural point of view, Omp-Pst1 shares the critical features of two acidic niches under L3, with E266 and D321 (equivalent to Omp-Pst2 E258 and D312) being the main contributors to niche I and II, respectively. Likewise, an aromatic residue is found at its L3 tip, *viz*. W114 (equivalent to W111 in Omp-Pst2). Yet, gating was not observed in Omp-Pst1 on the time-scale of our simulations, regardless of the directionality of the transmembrane potential. Accordingly, the HBN holding L3 attached to the barrel wall, W114-D321, remained unaffected throughout all simulations (Fig 5A). These observations are in faithful agreement with experimental data, which established a higher Vc for Omp-Pst1 than Omp-Pst2 (Table 1 and S11 Fig).

At V_TM_ < 0, binding of a K^+^ ion in niche I (~200 ns) occurs in monomer A, where it results in a conformational jump of L3 residues 115-AG-116 (equivalent to 112-GA-113 in Omp-Pst2) toward the extracellular side. In monomers B and C, however, neither binding of a K^+^ ion in niche I nor a conformational change in L3 is observed. Niche II meanwhile remains unoccupied in all three monomers (S12–S14 Figs). At V_TM_ > 0, binding of a K^+^ ion in niche I is observed in monomers A and B (after ~196 and ~254 ns, respectively), which precedes a conformational change in 115-AG-116 (after ~300 and ~500 ns, respectively). But again, niche II remains unoccupied in all three monomers (S15–S17 Figs). Thus at both V_TM_, and in all three Omp-Pst1 monomers, D321 remains covered by the side chains of L113 and W114. The tip of L3 is therefore kept fastened to the barrel wall, and W114 maintains its native, open-state conformation.

The discrepancy in the voltage sensitivity can be rationalized by a comparative analysis of Omp-Pst1 and Omp-Pst2 structures. In Omp-Pst2, the tip of L3 is attached to the barrel wall solely through hydrogen bonding of L110 and W110 main chain nitrogens to D312, whereas the presence of Q270, S280 and Y309 in Omp-Pst1 contributes three additional hydrogen bonds that manifestly increase the energy barrier of exposing the acidic niches (Fig 5B). The lower sensitivity to voltage of Omp-Pst1 (S18 Fig) is thus encoded in the HBN holding L3 attached to the barrel wall. Of note, we also observed asymmetry in HBN resilience in simulations of Omp-Pst1. At V_TM_ > 0, and not at V_TM_ < 0, the hydrogen bond between Y309 and V110 is disrupted at the end of the simulation. Therefore, Omp-Pst1 could gate faster at this V_TM_ (Fig 5C). Opposed asymmetries in voltage sensitivity were reported earlier for *E*. *coli* OmpF and PhoE [10].

## Discussion

This study reports on four 0.5 μs time-scale simulations conducted on the two porins from *P*. *stuartii*, *viz*. Omp-Pst1 and Omp-Pst2, at different transmembrane potentials. Under application of a negative transmembrane potential, we observed partial gating in one monomer of Omp-Pst2 (monomer B). Trajectory analysis revealed that a sequential changes on L3, including loop rearrangement and exposure of the hitherto hidden acidic niches, leads to protrusion of W11 side chain in the channel lumen. The presence of this large indol ring in the middle of the CZ hinders ion transit and results in the conductance of the monomer being decreased by ~50%. Based on curve fitting (*I*(*t*) = *a* × *e* ^*-b*×*t*^) to the calculated currents, we estimate that the time required for full closure is about tens of microseconds. Direct comparison from experiments under similar transmembrane potentials (V_TM_ = ±1 V) is not available, as lipid membranes do not withstand application of high voltages in experiments. However, we note that it was experimentally demonstrated that the time required for a bacterial porin to gate fully decreases exponentially as the applied voltage increases [6, 38]. Thus the estimated closing constant of Omp-Pst2 could be plausible. In the rest of our voltage-applied systems, stronger attachment of L3 loop onto the barrel wall was observed and the porins of study failed to undergo gating. We thus propose that the strength of this hydrogen bond network determines voltage sensitivity in the two porins from *P*. *stuartii*.

Based on our simulations, the most critical residues for porins (Omp-Pst2/1) gating are the acidic niches residues E258/E266 and D312/D321, on the one hand, and W111/W114 on the other hand. Sequence alignment with *E*. *coli* porins of known structures shows good conservation of these residues. First, all *E*. *coli* porins feature an aspartic acid at the position equivalent to D312 in Omp-Pst2 (niche II; *viz*. D312, D315 and D302 in OmpF, OmpC and PhoE, respectively). While E258 is not strictly conserved in terms of sequence position, negatively charged residues emanating from the barrel wall are found at other locations behind the L3 loop in *E*. *coli* porins, where they could contribute to an equivalent of niche I (E296 in OmpF; E260 and D299 in OmpC; E248 and D287 in PhoE) (S19B and S19C Fig). Also, an aromatic residue is found at the L3 tip of all *E*. *coli* porins (F118, F110 and F111 in OmpF, OmpC and PhoE, respectively) (S19A Fig). That residues critical for the voltage-gating of Omp-Pst2 are highly conserved in *E*. *coli* porins suggests that our gating model could apply to them as well. Furthermore, the lower sensitivity to transmembrane potential of OmpF, OmpC and PhoE (~155, ~185 and ~135 mV, respectively) correlates with the higher stabilization of their L3 loop onto the barrel wall. *E*. *coli* porins indeed all feature an additional glutamic acid at the L3 tip (E117, E109 and E110 in OmpF, OmpC and PhoE, respectively), which reinforces the attachment of their L3 tip to the barrel wall by one (Y22 in PhoE) or two hydrogen bonds (Y22/Y22 and Y310/Y313 in OmpF/OmpC). Thus, our proposal that it is the strength of the HBN attaching the tip of L3 onto the barrel wall that determines the likelihood of gating—and thus the sensitivity to voltage—is supported by examination of *E*. *coli* porins structures and by their Vc.

Further support to our proposed model for gating comes from mutagenesis data gathered on OmpF, which, similar to Omp-Pst2, is cation-selective. Replacement of acidic residues L3 (E117C) and niches residues (D312N, E296L, E296A and E296Q) by neutral ones, or stabilization of L3 by disulphide-bridge tethering onto the barrel wall (between E117C and A333C) [19, 22] both leads to increased Vc in OmpF. Reversely, replacement of basic residues in the anti L3 region (K16A, K16D, R42C, R82C, R132A, and R132D) [17, 18] leads to decreased Vc, as expected from a heavier trafficking of cations at the CZ—and thus an easier binding behind L3. That disulphide-tethering of L3 onto the barrel-wall does not suppress gating but merely increases the required voltage [20] is in accordance with our observation that gating does not require major conformation changes in L3, but a mere protrusion of an aromatic side chain in the channel lumen (here, W111 from Omp-Pst2). It needs to be acknowledged that fitting into our gating model mutagenesis data from anion-selective PhoE is less straightforward. While the critical residues are conserved (S19D Fig), mutagenesis data are pointing to the opposite direction, as discussed earlier [10, 15]. Mutation of residues involved in the attachment of the L3 tip to the barrel wall (E110C) or in the constitution of the acidic niche under L3 (E302C) indeed induce a decrease in Vc, while mutations of anti-L3 residues (R37C, R75C, R37C/R75C, K18C) all provoke an increase in Vc [15]. We believe that similar calculations need to be conducted on PhoE to provide an atomic level understanding of its possibly peculiar gating behaviour.

A tormenting question is whether voltage gating is a mere experimental artefact or underlies a functionally relevant regulatory mechanism. In early days, it was proposed that voltage gating could be a means by which porins that are mistakenly inserted into the inner membrane keep in the closed state [39, 40], because the Donnan potential of the outer membrane is ≤ -30 mV [39], while the inner membrane displays a transmembrane potential of about 160–200 mV (*i*.*e*. a value close to the Vc of most porins). Yet, the observation that Omp-Pst2 displays a strong propensity to gate (low Vc) suggests that this porin could, in the physiological context, rest in the gated state. That the gating propensity is asymmetric, and that this asymmetry correlates with the transportation of cations in an unfavourable direction—*i*.*e*. against the gradient of transmembrane potential—furthermore raises the question as to whether voltage gating conceals a role, for this porin, in the regulation of the cationic content of the periplasm. In humans, the primary habitat of *P*. *stuartii*, is the urinary tract, where the ammonium (NH4^+^) concentration is high. As *P*. *stuartii* features a urease activity that degrades urea into ammonia and carbonate, the higher propensity of Omp-Pst2 to transport cations from the intracellular to the extracellular side could participate in cleansing the bacterial periplasm of abounding cations. That Omp-Pst2 display a strong propensity to gate when the cationic flux occurs from the extracellular to the intracellular side furthermore suggests an active participation in the regulation of cationic fluxes across the outer-membrane. As new contributions of porins to bacterial development are discovered [41, 42], the functional meaning of voltage gating requires to be re-visited. In this context, molecular dynamics simulations hold the promise of allowing functional insights at the atomic level of resolution, as shown by the present work.

## Methods

### System setup

The crystal structure of Omp-Pst1 and Omp-Pst2 were solved at 3.3 Å and 2.2 Å respectively (PDB code 4D64 and 4D65 respectively). The crystal packing revealed an organization as dimers of trimers, *viz*. two symmetric, face-to-face trimmers. Chain A, B, and C of the crystal structures were taken out and used as starting models for the simulations. To create the lipid bilayer whereto embed our porins during the simulations, we used dimyristoylphosphatidylcholine (DMPC), as this lipid is of adequate length to model the thickness of a bacterial outer-membrane and has been used in a number of simulations studies on other bacterial porins [25, 43]. The bacterial outer-membrane is asymmetric, featuring lipopolysaccharides (LPS) on its extracellular leaflet. In our simulations, we did not use LPS, given that both experimental data collected on Omp-Pst1 and Omp-Pst2 reconstituted in LPS-containing bilayers and studies on other porins [44] established that the translocation properties of porins are not influenced by the presence of LPS. The trimeric structure of Omp-Pst1 and Omp-Pst2 were thus inserted into a pre-equilibrated lipid bilayer consisting of 512 DMPC by using the Perl script InflateGRO [45]. After the insertion, 58 DMPC were deleted, on the basis of steric clashes with the protein. In the final configuration, the area per lipid (APL) reached 60 Å^2^. Lipid-embedded Omp-Pst1 and Omp-Pst2 were then wrapped with 51,943 and 51,976 water molecules, respectively, in a cubic simulation box of 13.7 × 13.7 × 13.7 nm^3^. Potassium and chloride ions were added to reach a salt concentration of 1 M, and adjusted to provide neutral simulation systems. The standard protonation state at neutral pH was used for charged residues.

### Molecular dynamics simulation

Molecular dynamics simulations were performed using GROMACS 4.5 package [46], and all-atom CHARMM force field for proteins [47] and lipids [48]. The TIP3P model [49] was used for water molecules. In all simulations, periodic boundary conditions were used in x, y and z directions. Electrostatic interactions were computed using the particle mesh Ewald (PME) method [50]. A Fourier spacing of 0.11 nm was used to avoid spurious drifts in the center of mass of the system [28, 30]. The LINCS method [51] was used to restrain bond lengths, allowing integration steps of 2 fs and updating of the neighbor list every 5 fs (cut-off distance of 1.2 nm). Lennard-Jones and Coulomb cut-off distances were set to 1.4 and 1.2 nm, respectively.

To prepare the simulation systems, we used the following procedure. First, the initial configurations of the lipid-embedded Omp-Pst1 and Omp-Pst2 were optimized by four steps of energy minimization, during which positional restrains were imposed on i/ all none-hydrogen atoms, ii/ main-chain atoms, iii/ Cα atoms and iv/ no atoms. Thereby, maximum forces lesser than 100 kJ.mol^-1^.nm^-1^ was attained. The two systems were then thermalized to 310 K in six steps of NPT ensemble, each lasting 500 ps. In the NPT ensembles, the pressure was kept constant at 1 bar independently on the x-y plane (containing the lipid bilayer) and the z-axis direction (normal to the lipid bilayer) by semi-isotropic coupling to a Parrinello-Rahman barostat with τ_P_ = 1.0 ps and a compressibility of 4.6x10^-5^ bar [52], while the temperature was maintained at the target temperatures (50 K, 100 K, 150 K, 200 K, 250 K, and 310 K) by weakly (τ_T_ = 0.1 ps) coupling lipids, protein and solvent separately to a V-rescale thermostat [53]. Each system was then subjected to another 1 ns NVT ensemble at 310 K. After equilibration, the two systems were coupled to a homogenous electrostatic field E aligned with the z-axis, allowing for the simulations with an artificial transmembrane potential (E = V_TM_/L_Z_) of either +1 or -1 V (extracellular to intracellular). As for controls, we also subjected the Omp-Pst1 and Omp-Pst2 simulation systems to NVT ensembles in the absence of an electrostatic field (V_TM_ = 0 V). Simulations at V_TM_ = 0 V were carried out for 100 ns while those at V_TM_ ≠ 0 for 500 ns each. A recapitulation of the simulations is given in S1 Table.

### Data analysis

Net ion permeation events and residency times within the channels were calculated using g_flux tool [54]. Ions that bound in niche I and II and the occupancy of the two niches were counted using the GROMCAS tool of g_select. Ions were required to move from one side of the lipid to the other to count as a complete permeation event. The selectivity and conductance of Omp-Pst1 and Omp-Pst2 were determined by calculating the current-voltage relationship under different applied electric fields. In each simulation, the current contributed either by K^+^ or Cl^-^ ions was determined through linear regression of the net ion crossing at every time interval of Δ*t* = 50 ns. At each voltage, the total current was the sum of K^+^ and Cl^-^ currents, and conductances were calculated as the slope of the current-voltage curves [55]. Voltage-specific ion selectivity was calculated by using the ratio of K^+^ and Cl^-^ current at each voltage. Simulated ion diffusion coefficients were calculated from our 100 ns simulations at V_TM_ = 0. Diffusion coefficients obtained for K^+^ and Cl^-^ were 1.83 cm^2^/s and 1.86 cm^2^/s in the simulation of Omp-Pst1, and 1.72 cm^2^/s and 1.95 cm^2^/s in the simulation of Omp-Pst2. The ratio of the experimentally determined diffusion coefficients (1.96 cm^2^/s for K^+^ and 2.02 cm^2^/s for Cl^-^ [56]) to the calculated ones was used to get the corrected conductance. In practice, the K^+^ and Cl^-^ currents were scaled by the ratio before calculation of the slope of the I-V curves. Ion densities within the channels (S4 Fig) were calculated using the density calculation module in MDAnalysis tool [57]. Hydrogen-bonding network analysis was performed using VMD (Figs 4A and 5A). Sequence alignments were performed using CLUSTALW. PhoE residue numbering was adjusted to fit the nomenclature used in previous papers [10, 15, 18].

### Electrophysiology experiment setup

Planar lipid bilayers were formed according to the monolayer technique of Montal and Mueller [58]. The bilayer was formed across a hole that was about 50 μm in diameter in a 25 μm thick polytetrafluoroethylene (PTFE) film. A lipid bilayer was prepared by spreading 1 μL of a 5 mg/mL solution of 1,2-diphytanoyl-sn-glycero-3-phosphocholine in a solvent mixture of n-pentane in the aperture. Ag/AgCl electrodes were used to detect the ionic currents. The electrode on the *cis* side of the cell was grounded, whereas the other one on the *trans* side was connected to the headstage of an Axopatch 200B amplifier. Purified detergent-solubilized porins (1 ng/mL) were added to the *cis* side of the chamber in presence of 1M KCl, 20mM PO_4_ pH 4 and inserted into the bilayer membrane by applying a 150–200 mV voltage. The recordings were made after diluting the same chamber with 1M KCl, 10mM HEPES pH 7.

## Supporting Information

S1 Fig RMS fluctuation. The root mean square fluctuation of protein main-chain atoms was calculated from the 500 ns trajectory in the voltage-applied systems and 100 ns in the non-voltage ones. Blue, green and red lines represent the values in monomer A, B and C respectively. Chain assignment was taken from the crystal structures. The L3 region is highlighted out in gray.
(TIF)

S2 Fig Fluctuation of extracellular loops. The movements of extracellular loops were projected to the 1^st^ eigenvector of the whole protein in each system. Snapshots were taken every 100 ns.
(TIF)

S3 Fig Net ion permeation. The cumulative number of K^+^ or Cl^-^ crossing events in each monomer is traced as function of time. In each panel, blue, green and red lines represent crossing events in monomer A, B and C respectively.
(TIF)

S4 Fig Ion density maps within the channel. The ion density was averaged from the first 100 ns simulations. Heavy ion densities are observed in the constriction zone of Omp-Pst1 and both constriction zone and extracellular vestibule of Omp-Pst2. The red and blue contour surfaces are the Cl- and the K+ ions density of 0.003 Å^-3^ respectively. Each β-barrel denotes one of the three monomers.
(TIF)

S5 Fig Currents in Omp-Pst2 monomer B at negative voltage. Blue and red dots denote values for K^+^ and Cl^-^ respectively. Black dots are the totally currents of K^+^ and Cl^-^ combined. Currents were calculated every 50 ns.
(TIF)

S6 Fig L3 movements of Omp-Pst2 monomer C at negative voltage. (A) Snapshots of L3 tip. In each snapshots, residues W104 to D117 are shown in cartoon; W111, D114, E258 and D312 in sticks; and K^+^ within 3.5 Å of the tip in blue spheres. The acidic niche I (E258) and niche II (D312) are highlighted in gray. (B) W111 movements taken every 50 ns. The RMSD of 112-AG-113 (C), the phi/psi angle of W111 (D) and the RMSD of W111 (E) are laid out to show the sequential events of exposure and binding of the two niches under L3 (F).
(TIF)

S7 Fig L3 movements of Omp-Pst2 monomer A at negative voltage. (A) Snapshots of L3 tip. In each snapshots, residues W104 to D117 are shown in cartoon; W111, D114, E258 and D312 in sticks; and K^+^ within 3.5 Å of the tip in blue spheres. The acidic niche I (E258) and niche II (D312) are highlighted in gray. (B) W111 movements taken every 50 ns. The RMSD of 112-AG-113 (C), the phi/psi angle of W111 (D) and the RMSD of W111 (E) are laid out to show the sequential events of exposure and binding of the two niches under L3 (F).
(TIF)

S8 Fig L3 movements of Omp-Pst2 monomer A at positive voltage. (A) Snapshots of L3 tip. In each snapshots, residues W104 to D117 are shown in cartoon; W111, D114, E258 and D312 in sticks; and K^+^ within 3.5 Å of the tip in blue spheres. The acidic niche I (E258) and niche II (D312) are highlighted in gray. (B) W111 movements taken every 50 ns. The RMSD of 112-AG-113 (C), the phi/psi angle of W111 (D) and the RMSD of W111 (E) are laid out to show the sequential events of exposure and binding of the two niches under L3 (F).
(TIF)

S9 Fig L3 movements of Omp-Pst2 monomer B at positive voltage. (A) Snapshots of L3 tip. In each snapshots, residues W104 to D117 are shown in cartoon; W111, D114, E258 and D312 in sticks; and K^+^ within 3.5 Å of the tip in blue spheres. The acidic niche I (E258) and niche II (D312) are highlighted in gray. (B) W111 movements taken every 50 ns. The RMSD of 112-AG-113 (C), the phi/psi angle of W111 (D) and the RMSD of W111 (E) are laid out to show the sequential events of exposure and binding of the two niches under L3 (F).
(TIF)

S10 Fig L3 movements of Omp-Pst2 monomer C at positive voltage. (A) Snapshots of L3 tip. In each snapshots, residues W104 to D117 are shown in cartoon; W111, D114, E258 and D312 in sticks; and K^+^ within 3.5 Å of the tip in blue spheres. The acidic niche I (E258) and niche II (D312) are highlighted in gray. (B) W111 movements taken every 50 ns. The RMSD of 112-AG-113 (C), the phi/psi angle of W111 (D) and the RMSD of W111 (E) are laid out to show the sequential events of exposure and binding of the two niches under L3 (F).
(TIF)

S11 Fig Gating of Omp-Pst1 and Omp-Pst2 in planar lipid bilayer. Representative ion current trace and their corresponding histograms show three-step voltage gating of Omp-Pst1 at -199 mV (A) and Omp-Pst2 at -50 mV (B). Buffer conditions: 1M KCl, 10 mM HEPES at pH 7.
(TIF)

S12 Fig L3 movements of Omp-Pst1 monomer A at negative voltage. (A) Snapshots of L3 tip. In each snapshots, residues W107 to D120 are shown in cartoon; W114, D117, E266 and D321 in sticks; and K^+^ within 3.5 Å of the tip in blue spheres. The acidic niche I (E266) and niche II (D321) are highlighted in gray. (B) W111 movements taken every 50 ns. The RMSD of 115-AG-116 (C), the phi/psi angle of W114 (D) and the RMSD of W114 (E) are laid out to show the sequential events of exposure and binding of the two niches under L3 (F).
(TIF)

S13 Fig L3 movements of Omp-Pst1 monomer B at negative voltage. (A) Snapshots of L3 tip. In each snapshots, residues W107 to D120 are shown in cartoon; W114, D117, E266 and D321 in sticks; and K^+^ within 3.5 Å of the tip in blue spheres. The acidic niche I (E266) and niche II (D321) are highlighted in gray. (B) W111 movements taken every 50 ns. The RMSD of 115-AG-116 (C), the phi/psi angle of W114 (D) and the RMSD of W114 (E) are laid out to show the sequential events of exposure and binding of the two niches under L3 (F).
(TIF)

S14 Fig L3 movements of Omp-Pst1 monomer C at negative voltage. (A) Snapshots of L3 tip. In each snapshots, residues W107 to D120 are shown in cartoon; W114, D117, E266 and D321 in sticks; and K^+^ within 3.5 Å of the tip in blue spheres. The acidic niche I (E266) and niche II (D321) are highlighted in gray. (B) W111 movements taken every 50 ns. The RMSD of 115-AG-116 (C), the phi/psi angle of W114 (D) and the RMSD of W114 (E) are laid out to show the sequential events of exposure and binding of the two niches under L3 (F).
(TIF)

S15 Fig L3 movements of Omp-Pst1 monomer A at positive voltage. (A) Snapshots of L3 tip. In each snapshots, residues W107 to D120 are shown in cartoon; W114, D117, E266 and D321 in sticks; and K^+^ within 3.5 Å of the tip in blue spheres. The acidic niche I (E266) and niche II (D321) are highlighted in gray. (B) W111 movements taken every 50 ns. The RMSD of 115-AG-116 (C), the phi/psi angle of W114 (D) and the RMSD of W114 (E) are laid out to show the sequential events of exposure and binding of the two niches under L3 (F).
(TIF)

S16 Fig L3 movements of Omp-Pst1 monomer B at positive voltage. (A) Snapshots of L3 tip. In each snapshots, residues W107 to D120 are shown in cartoon; W114, D117, E266 and D321 in sticks; and K^+^ within 3.5 Å of the tip in blue spheres. The acidic niche I (E266) and niche II (D321) are highlighted in gray. (B) W111 movements taken every 50 ns. The RMSD of 115-AG-116 (C), the phi/psi angle of W114 (D) and the RMSD of W114 (E) are laid out to show the sequential events of exposure and binding of the two niches under L3 (F).
(TIF)

S17 Fig L3 movements of Omp-Pst1 monomer C at positive voltage. (A) Snapshots of L3 tip. In each snapshots, residues W107 to D120 are shown in cartoon; W114, D117, E266 and D321 in sticks; and K^+^ within 3.5 Å of the tip in blue spheres. The acidic niche I (E266) and niche II (D321) are highlighted in gray. (B) W111 movements taken every 50 ns. The RMSD of 115-AG-116 (C), the phi/psi angle of W114 (D) and the RMSD of W114 (E) are laid out to show the sequential events of exposure and binding of the two niches under L3 (F).
(TIF)

S18 Fig Gating of Omp-Pst2 at -20 mV in planar lipid bilayer. Upper panel: the ion current trace; Lower panel: the corresponding histograms of the current trace. Buffer conditions: 1M KCl, 10 mM HEPES at pH 7.
(TIF)

S19 Fig Sequence and structure conservation between Omp-Psts and the *E*. *coli* general diffusion porins. A) Sequence alignment of L3 of Omp-Pst1, Omp-Pst2, OmpF, OmpC and PhoE. Identical residues are highlighted in red and similar residues in gray. The residue that the blue triangle points out is the conserved aromatic residue at L3 tip. B) L3 tip of OmpF, OmpC and PhoE. The conserved aromatic residues are shown in sticks; the glutamic acid and the residue(s) it form hydrophone-bonding(s) with in sticks; and the main contributors of the acidic niches beneath L3 in sticks and highlighted in gray.
(TIF)

S1 Table System configurations. (DOCX)
