# Supplementary figures and images for: Understanding Voltage Gating of Providencia stuartii Porins at Atomic Level
Source: PLoS Comput Biol. 2015 May 8;11(5):e1004255. doi: 10.1371/journal.pcbi.1004255 (PMC4425685; doi:10.1371/journal.pcbi.1004255)

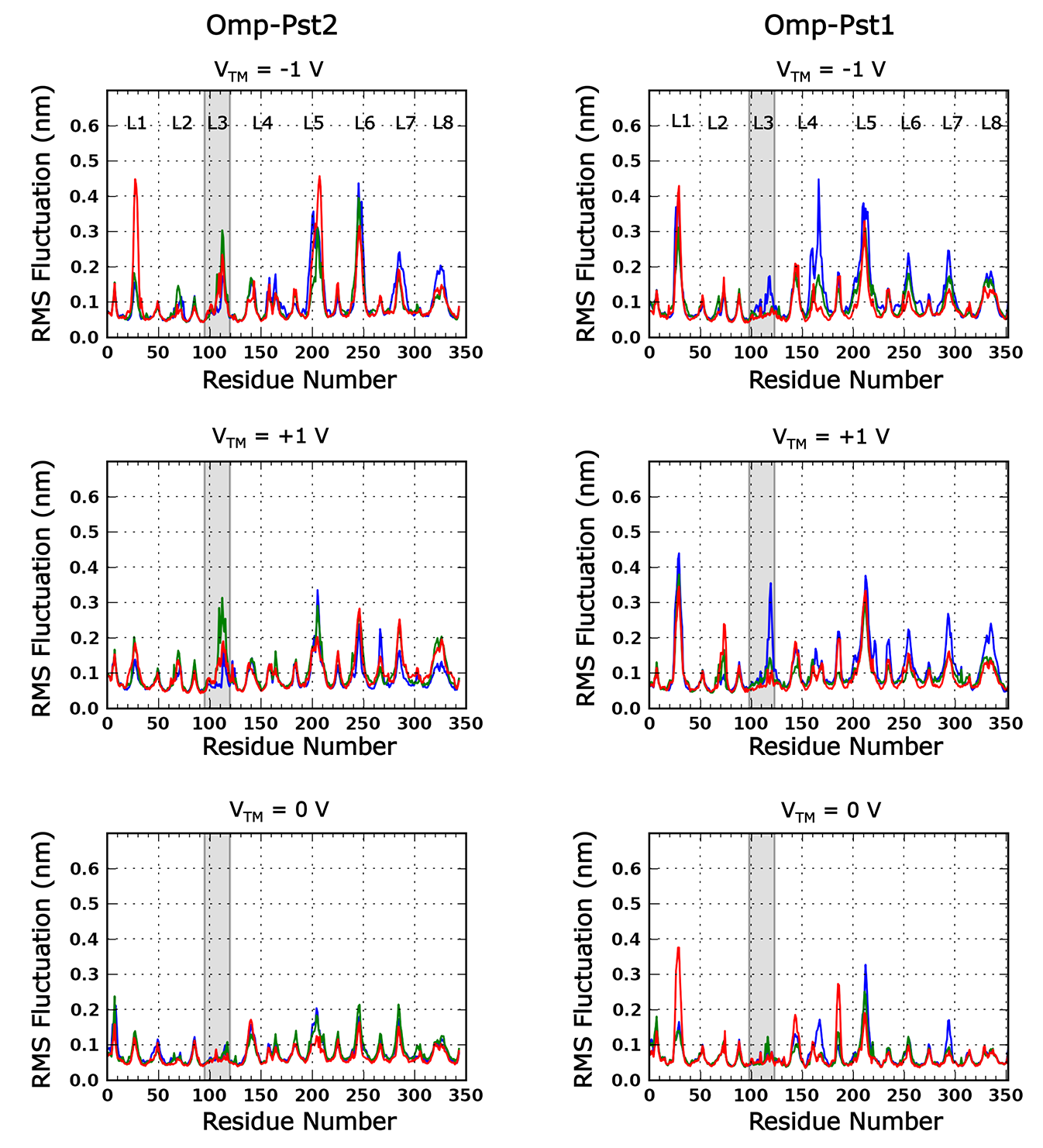

Supplement: S1 Fig — The root mean square fluctuation of protein main-chain atoms was calculated from the 500 ns trajectory in the voltage-applied systems and 100 ns in the non-voltage ones. Blue, green and red lines represent the values in monomer A, B and C respectively. Chain assignment was taken from the crystal structures. The L3 region is highlighted out in gray. (TIF) [file pcbi.1004255.s001.tif]

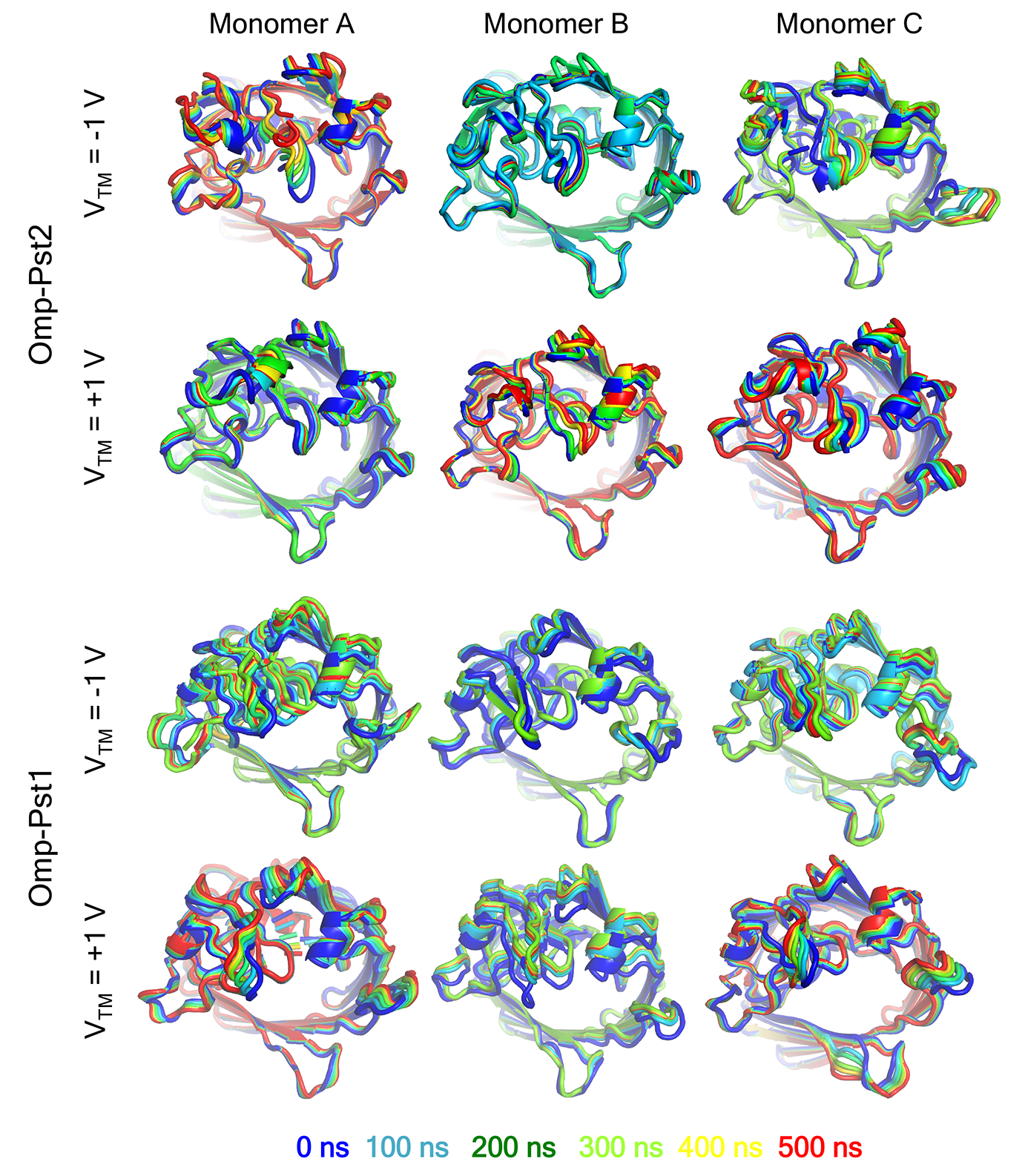

Supplement: S2 Fig — The movements of extracellular loops were projected to the 1st eigenvector of the whole protein in each system. Snapshots were taken every 100 ns. (TIF) [file pcbi.1004255.s002.tif]

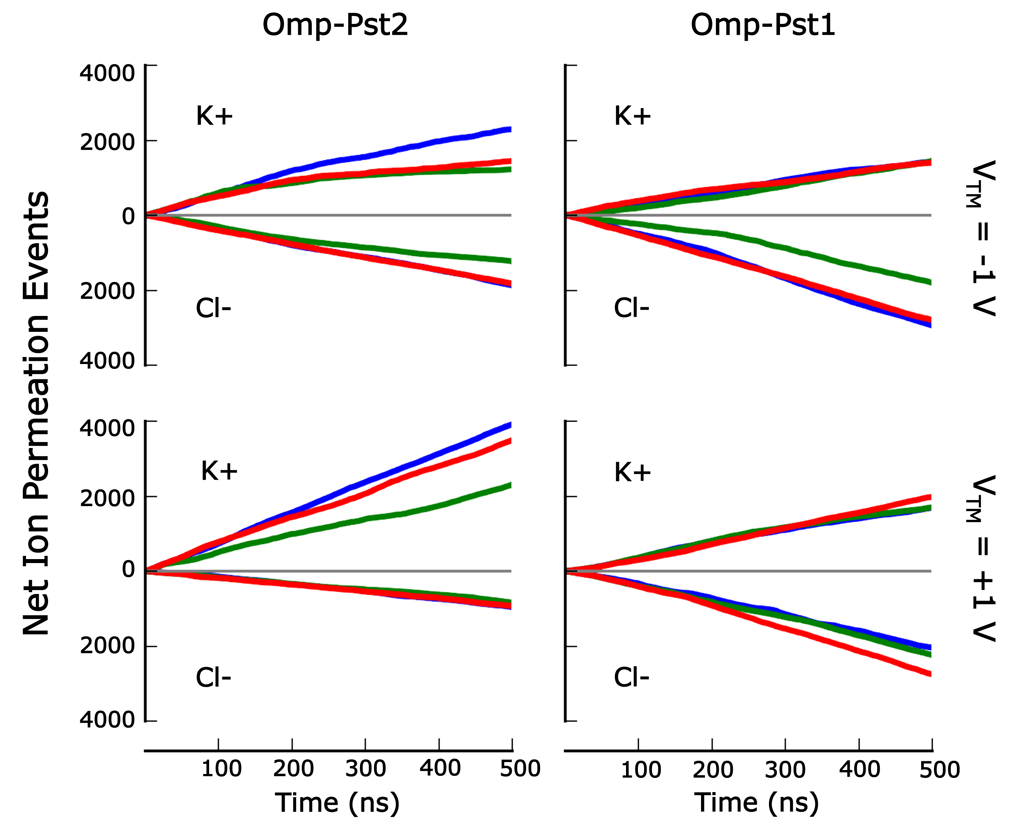

Supplement: S3 Fig — The cumulative number of K+ or Cl- crossing events in each monomer is traced as function of time. In each panel, blue, green and red lines represent crossing events in monomer A, B and C respectively. (TIF) [file pcbi.1004255.s003.tif]

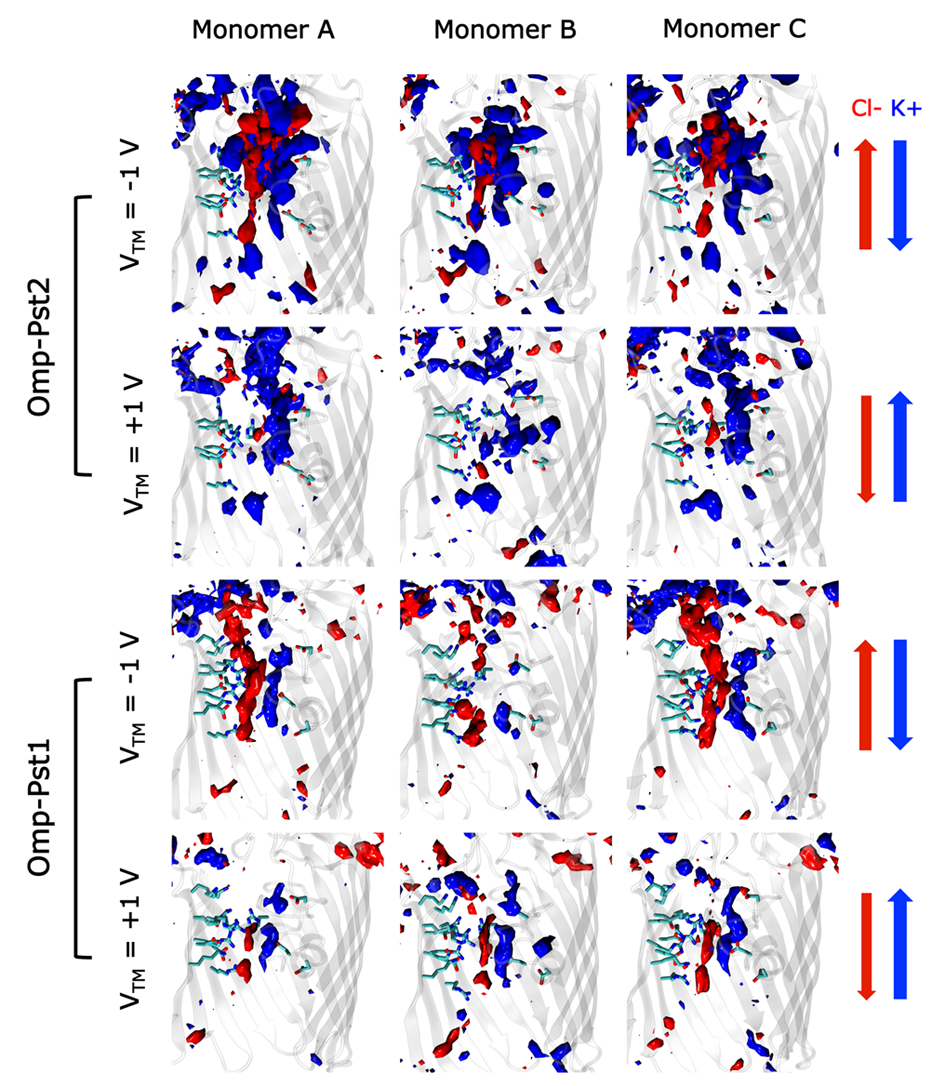

Supplement: S4 Fig — The ion density was averaged from the first 100 ns simulations. Heavy ion densities are observed in the constriction zone of Omp-Pst1 and both constriction zone and extracellular vestibule of Omp-Pst2. The red and blue contour surfaces are the Cl- and the K+ ions density of 0.003 Å-3 respectively. Each β-barrel denotes one of the three monomers. (TIF) [file pcbi.1004255.s004.tif]

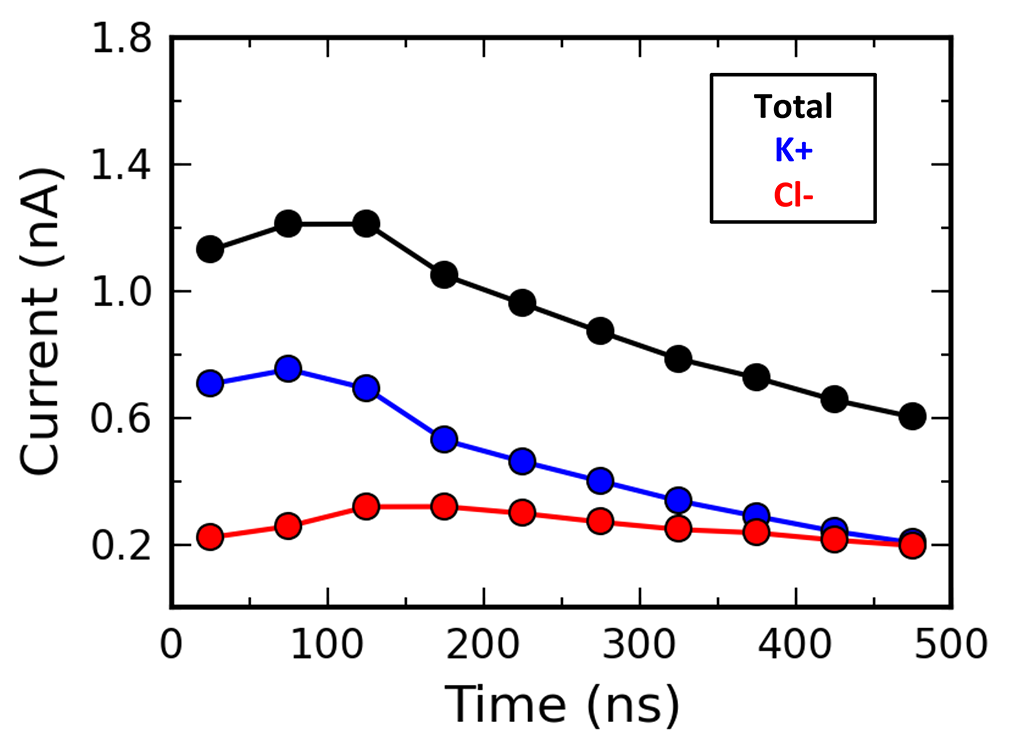

Supplement: S5 Fig — Blue and red dots denote values for K+ and Cl- respectively. Black dots are the totally currents of K+ and Cl- combined. Currents were calculated every 50 ns. (TIF) [file pcbi.1004255.s005.tif]

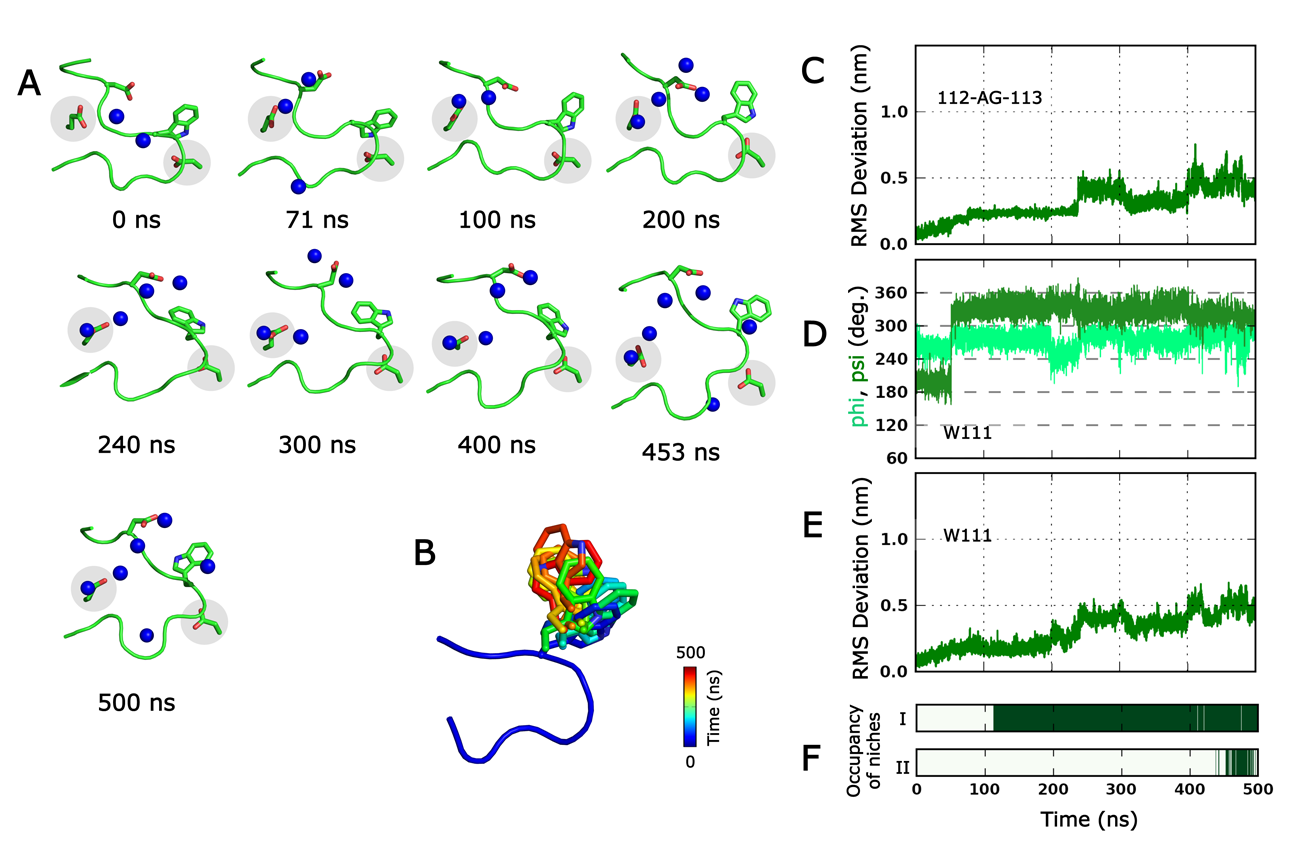

Supplement: S6 Fig — (A) Snapshots of L3 tip. In each snapshots, residues W104 to D117 are shown in cartoon; W111, D114, E258 and D312 in sticks; and K+ within 3.5 Å of the tip in blue spheres. The acidic niche I (E258) and niche II (D312) are highlighted in gray. (B) W111 movements taken every 50 ns. The RMSD of 112-AG-113 (C), the phi/psi angle of W111 (D) and the RMSD of W111 (E) are laid out to show the sequential events of exposure and binding of the two niches under L3 (F). (TIF) [file pcbi.1004255.s006.tif]

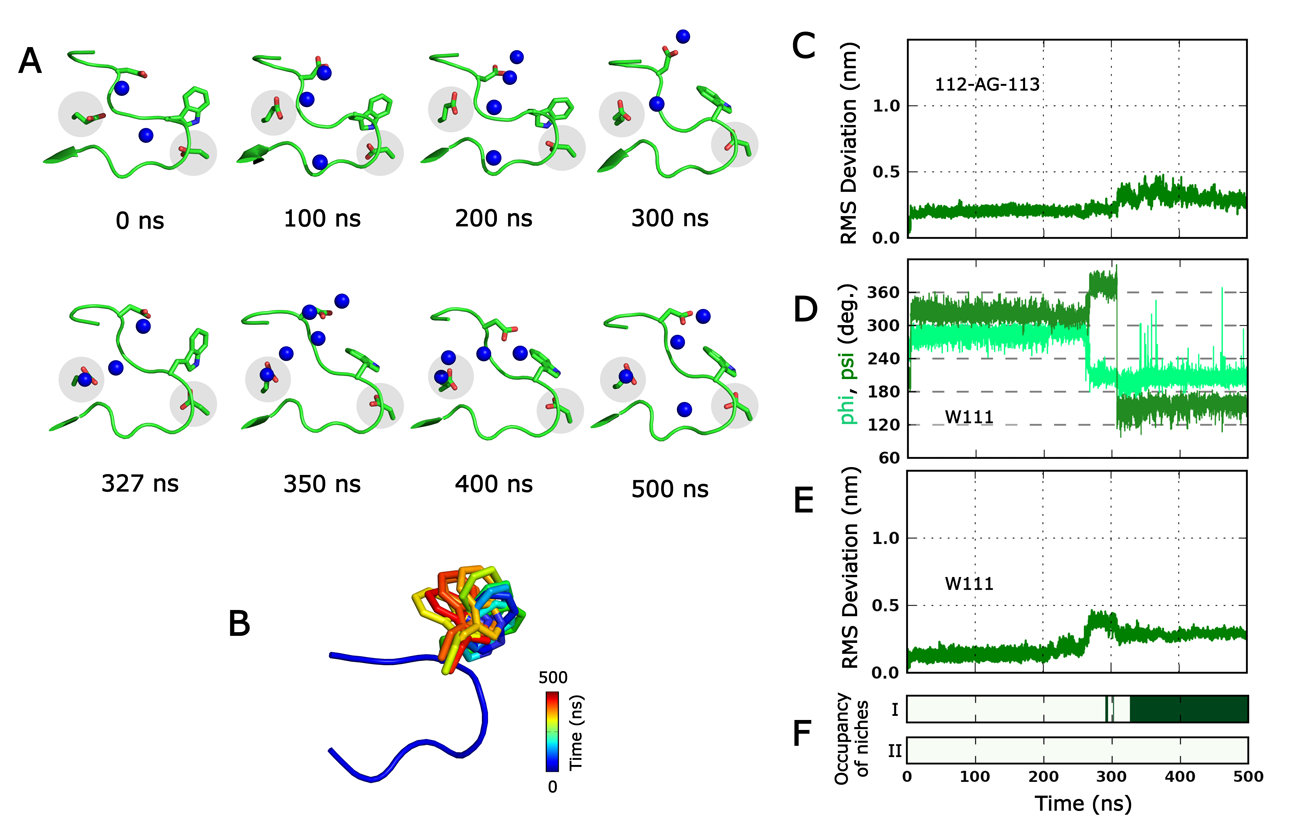

Supplement: S7 Fig — (A) Snapshots of L3 tip. In each snapshots, residues W104 to D117 are shown in cartoon; W111, D114, E258 and D312 in sticks; and K+ within 3.5 Å of the tip in blue spheres. The acidic niche I (E258) and niche II (D312) are highlighted in gray. (B) W111 movements taken every 50 ns. The RMSD of 112-AG-113 (C), the phi/psi angle of W111 (D) and the RMSD of W111 (E) are laid out to show the sequential events of exposure and binding of the two niches under L3 (F). (TIF) [file pcbi.1004255.s007.tif]

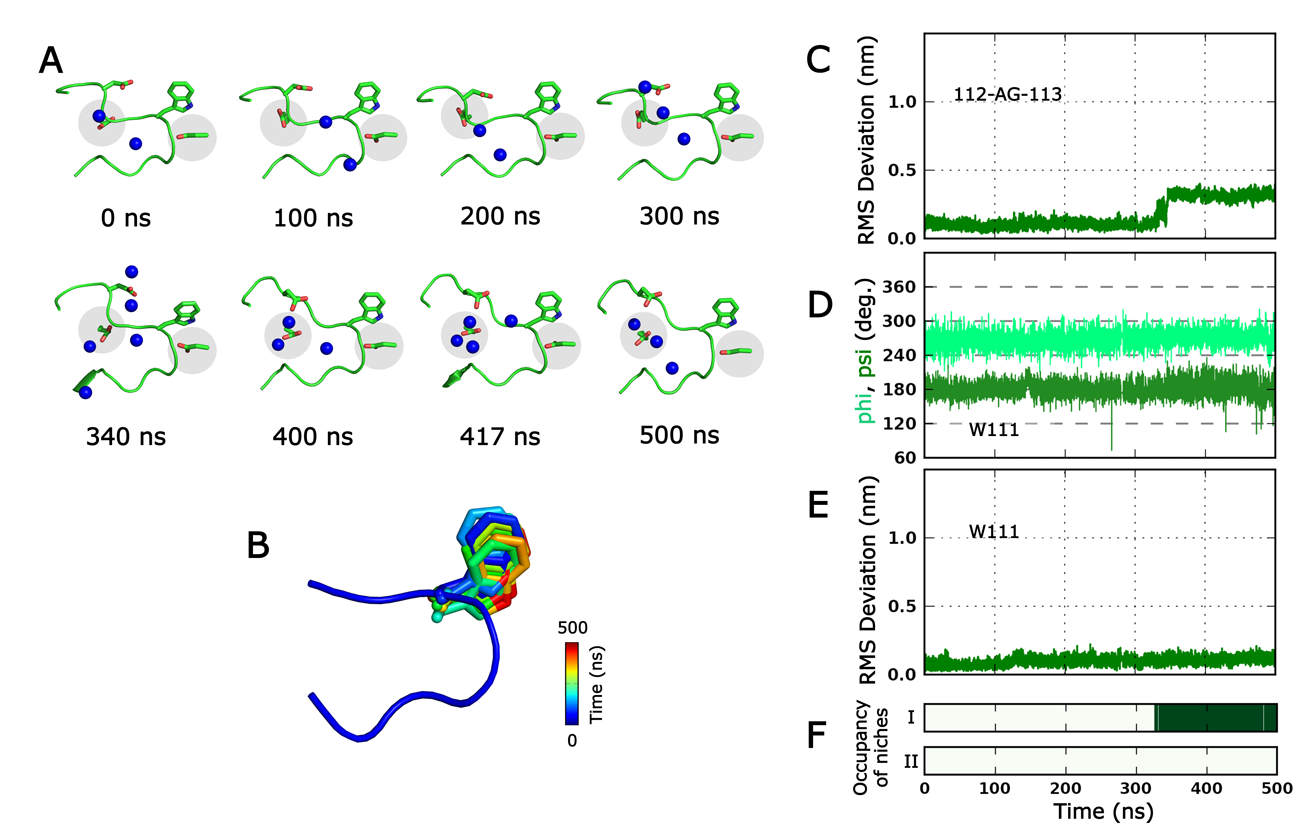

Supplement: S8 Fig — (A) Snapshots of L3 tip. In each snapshots, residues W104 to D117 are shown in cartoon; W111, D114, E258 and D312 in sticks; and K+ within 3.5 Å of the tip in blue spheres. The acidic niche I (E258) and niche II (D312) are highlighted in gray. (B) W111 movements taken every 50 ns. The RMSD of 112-AG-113 (C), the phi/psi angle of W111 (D) and the RMSD of W111 (E) are laid out to show the sequential events of exposure and binding of the two niches under L3 (F). (TIF) [file pcbi.1004255.s008.tif]

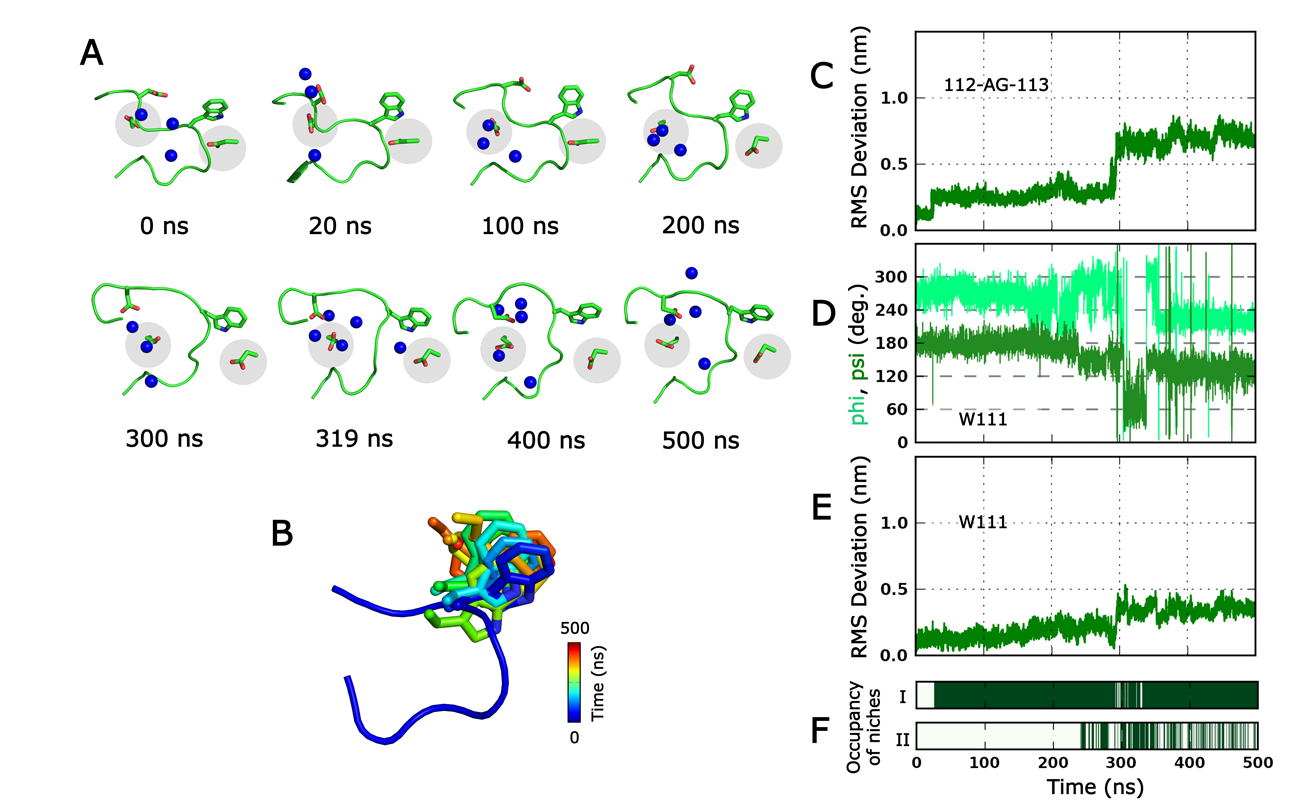

Supplement: S9 Fig — (A) Snapshots of L3 tip. In each snapshots, residues W104 to D117 are shown in cartoon; W111, D114, E258 and D312 in sticks; and K+ within 3.5 Å of the tip in blue spheres. The acidic niche I (E258) and niche II (D312) are highlighted in gray. (B) W111 movements taken every 50 ns. The RMSD of 112-AG-113 (C), the phi/psi angle of W111 (D) and the RMSD of W111 (E) are laid out to show the sequential events of exposure and binding of the two niches under L3 (F). (TIF) [file pcbi.1004255.s009.tif]

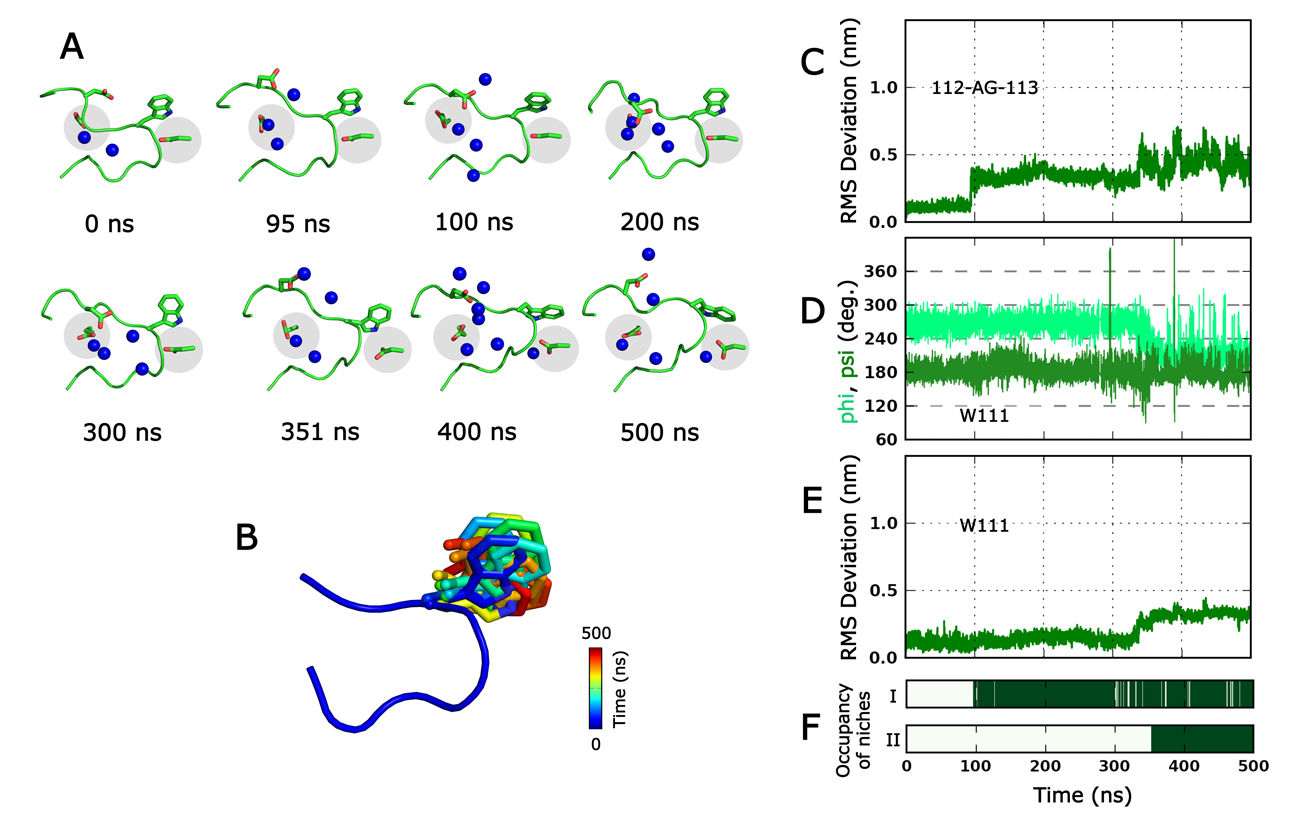

Supplement: S10 Fig — (A) Snapshots of L3 tip. In each snapshots, residues W104 to D117 are shown in cartoon; W111, D114, E258 and D312 in sticks; and K+ within 3.5 Å of the tip in blue spheres. The acidic niche I (E258) and niche II (D312) are highlighted in gray. (B) W111 movements taken every 50 ns. The RMSD of 112-AG-113 (C), the phi/psi angle of W111 (D) and the RMSD of W111 (E) are laid out to show the sequential events of exposure and binding of the two niches under L3 (F). (TIF) [file pcbi.1004255.s010.tif]

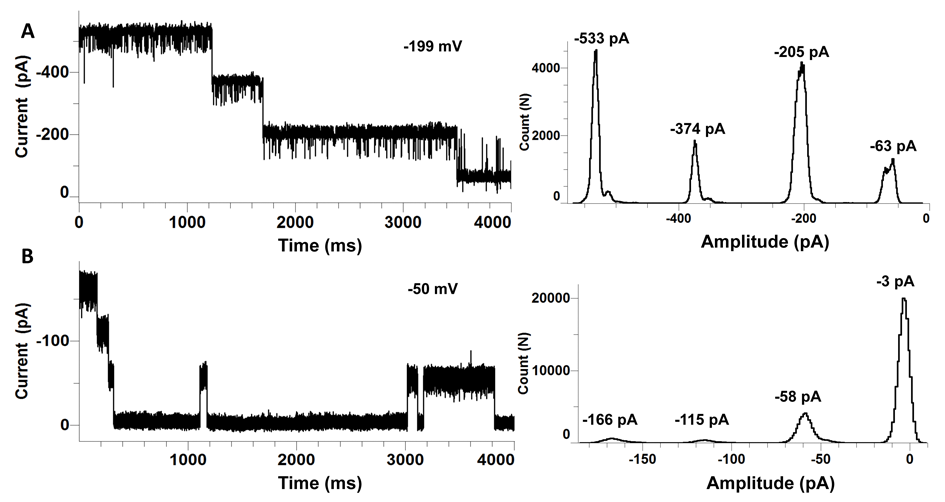

Supplement: S11 Fig — Representative ion current trace and their corresponding histograms show three-step voltage gating of Omp-Pst1 at -199 mV (A) and Omp-Pst2 at -50 mV (B). Buffer conditions: 1M KCl, 10 mM HEPES at pH 7. (TIF) [file pcbi.1004255.s011.tif]

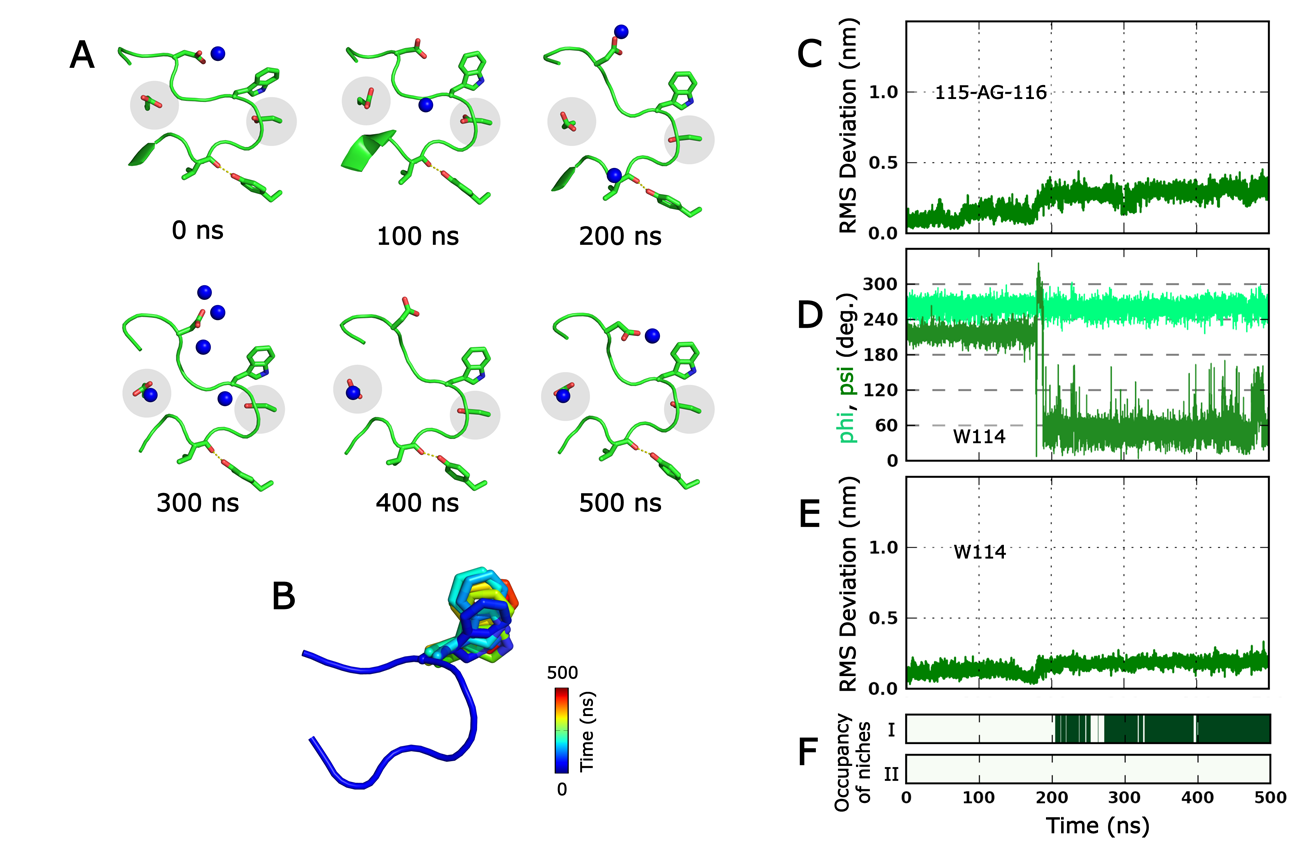

Supplement: S12 Fig — (A) Snapshots of L3 tip. In each snapshots, residues W107 to D120 are shown in cartoon; W114, D117, E266 and D321 in sticks; and K+ within 3.5 Å of the tip in blue spheres. The acidic niche I (E266) and niche II (D321) are highlighted in gray. (B) W111 movements taken every 50 ns. The RMSD of 115-AG-116 (C), the phi/psi angle of W114 (D) and the RMSD of W114 (E) are laid out to show the sequential events of exposure and binding of the two niches under L3 (F). (TIF) [file pcbi.1004255.s012.tif]

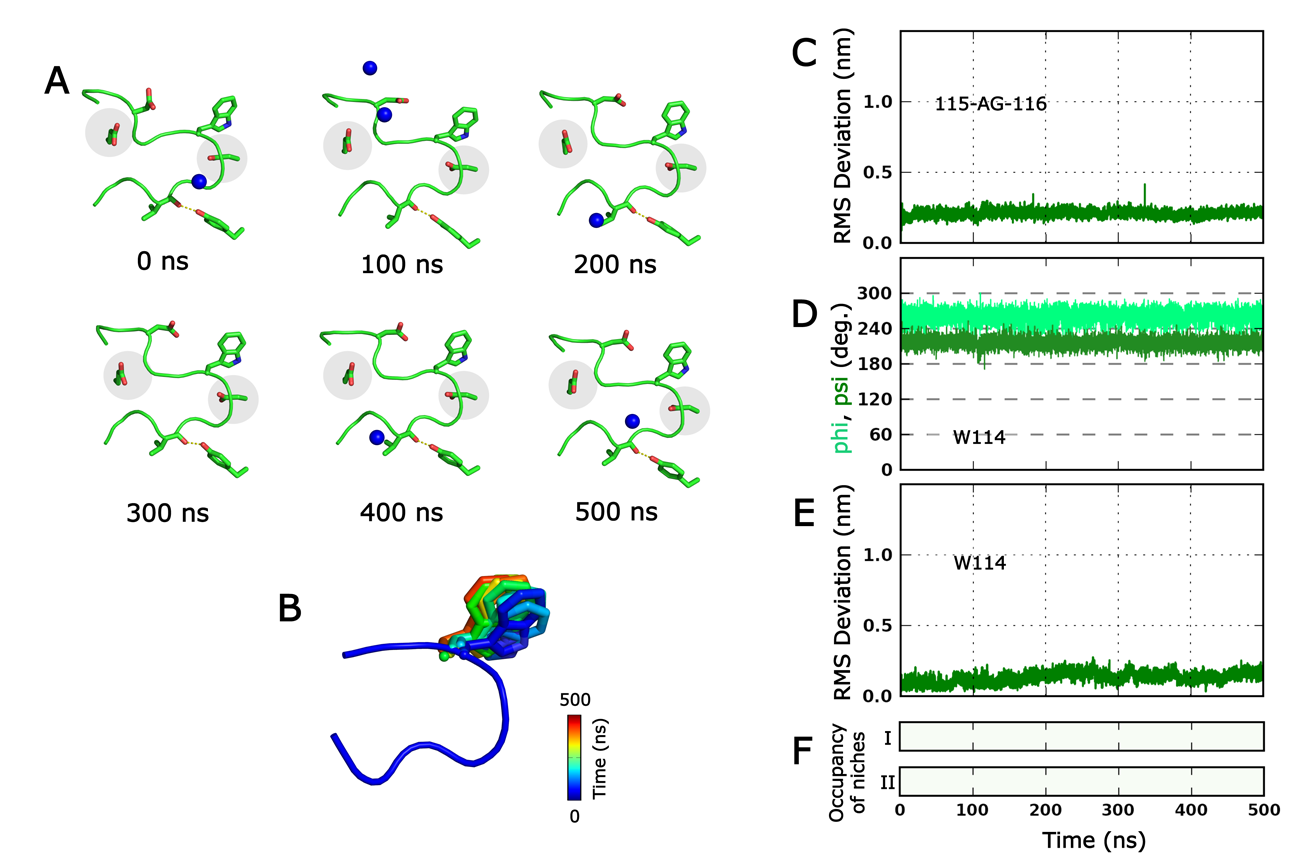

Supplement: S13 Fig — (A) Snapshots of L3 tip. In each snapshots, residues W107 to D120 are shown in cartoon; W114, D117, E266 and D321 in sticks; and K+ within 3.5 Å of the tip in blue spheres. The acidic niche I (E266) and niche II (D321) are highlighted in gray. (B) W111 movements taken every 50 ns. The RMSD of 115-AG-116 (C), the phi/psi angle of W114 (D) and the RMSD of W114 (E) are laid out to show the sequential events of exposure and binding of the two niches under L3 (F). (TIF) [file pcbi.1004255.s013.tif]

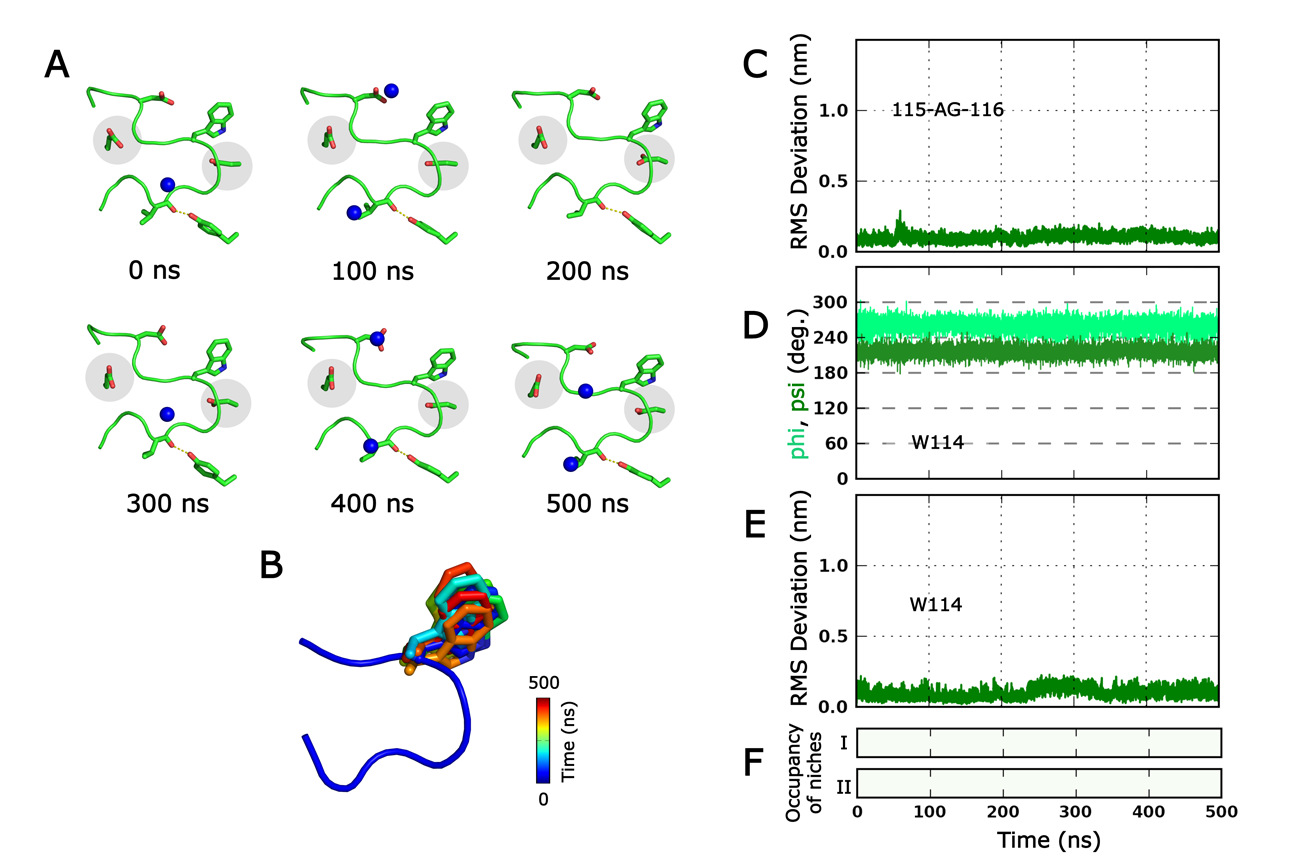

Supplement: S14 Fig — (A) Snapshots of L3 tip. In each snapshots, residues W107 to D120 are shown in cartoon; W114, D117, E266 and D321 in sticks; and K+ within 3.5 Å of the tip in blue spheres. The acidic niche I (E266) and niche II (D321) are highlighted in gray. (B) W111 movements taken every 50 ns. The RMSD of 115-AG-116 (C), the phi/psi angle of W114 (D) and the RMSD of W114 (E) are laid out to show the sequential events of exposure and binding of the two niches under L3 (F). (TIF) [file pcbi.1004255.s014.tif]

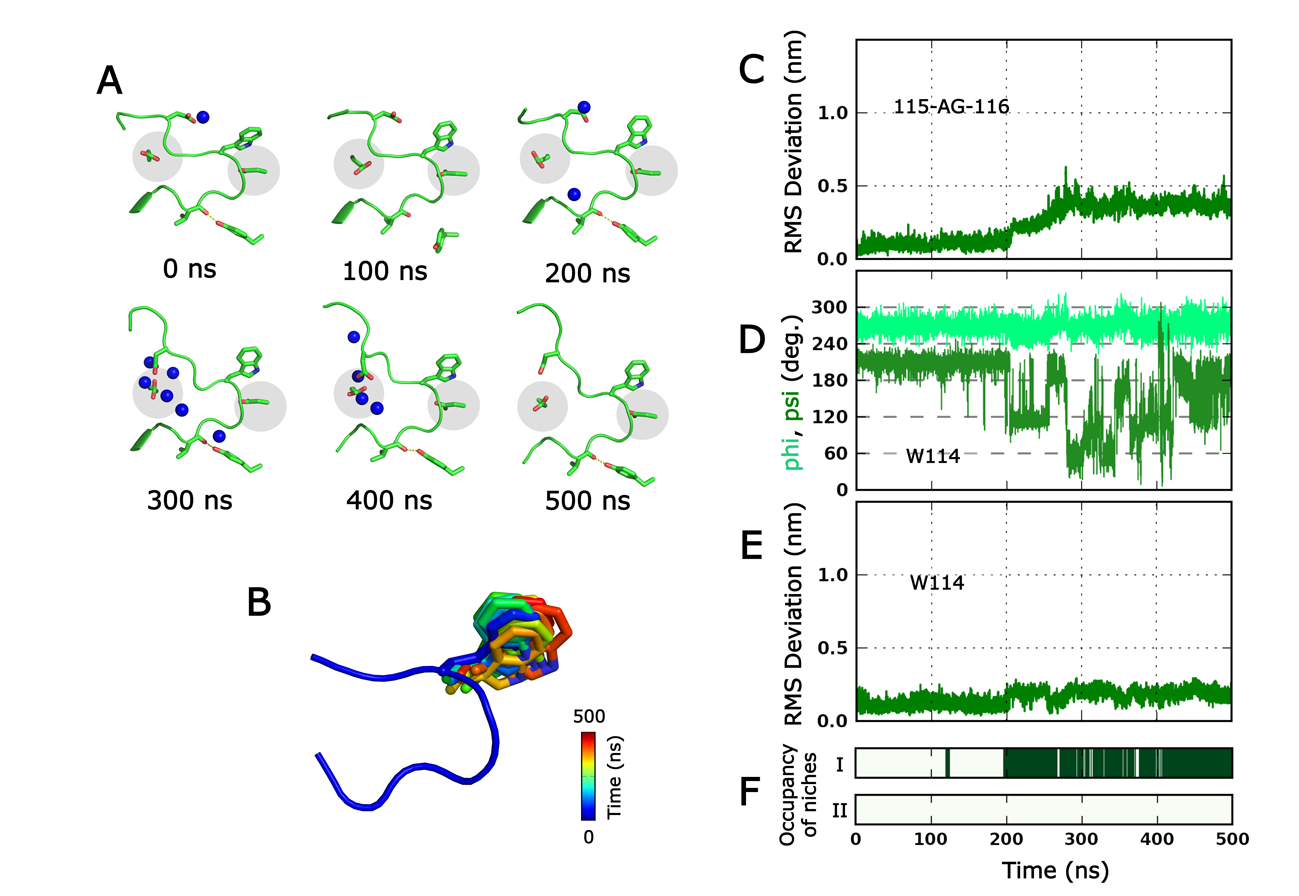

Supplement: S15 Fig — (A) Snapshots of L3 tip. In each snapshots, residues W107 to D120 are shown in cartoon; W114, D117, E266 and D321 in sticks; and K+ within 3.5 Å of the tip in blue spheres. The acidic niche I (E266) and niche II (D321) are highlighted in gray. (B) W111 movements taken every 50 ns. The RMSD of 115-AG-116 (C), the phi/psi angle of W114 (D) and the RMSD of W114 (E) are laid out to show the sequential events of exposure and binding of the two niches under L3 (F). (TIF) [file pcbi.1004255.s015.tif]

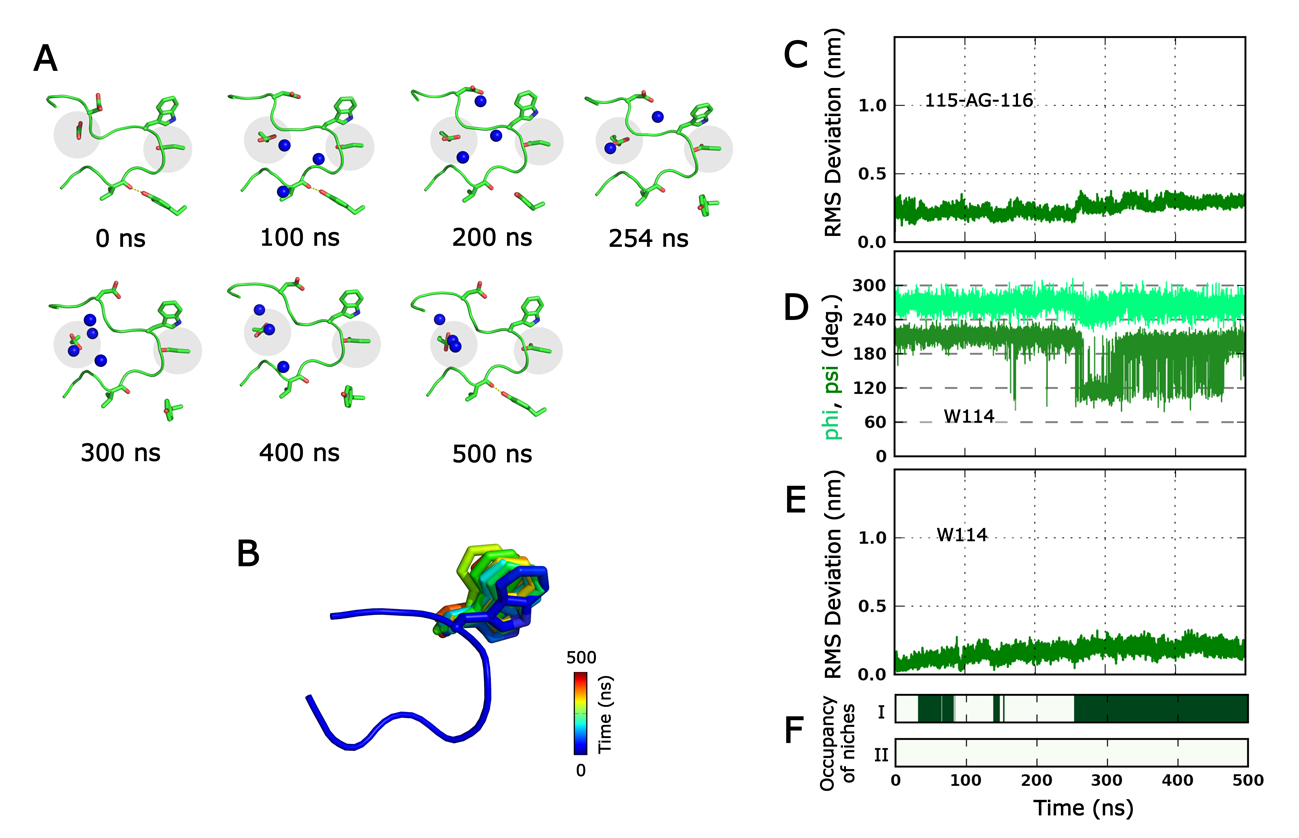

Supplement: S16 Fig — (A) Snapshots of L3 tip. In each snapshots, residues W107 to D120 are shown in cartoon; W114, D117, E266 and D321 in sticks; and K+ within 3.5 Å of the tip in blue spheres. The acidic niche I (E266) and niche II (D321) are highlighted in gray. (B) W111 movements taken every 50 ns. The RMSD of 115-AG-116 (C), the phi/psi angle of W114 (D) and the RMSD of W114 (E) are laid out to show the sequential events of exposure and binding of the two niches under L3 (F). (TIF) [file pcbi.1004255.s016.tif]

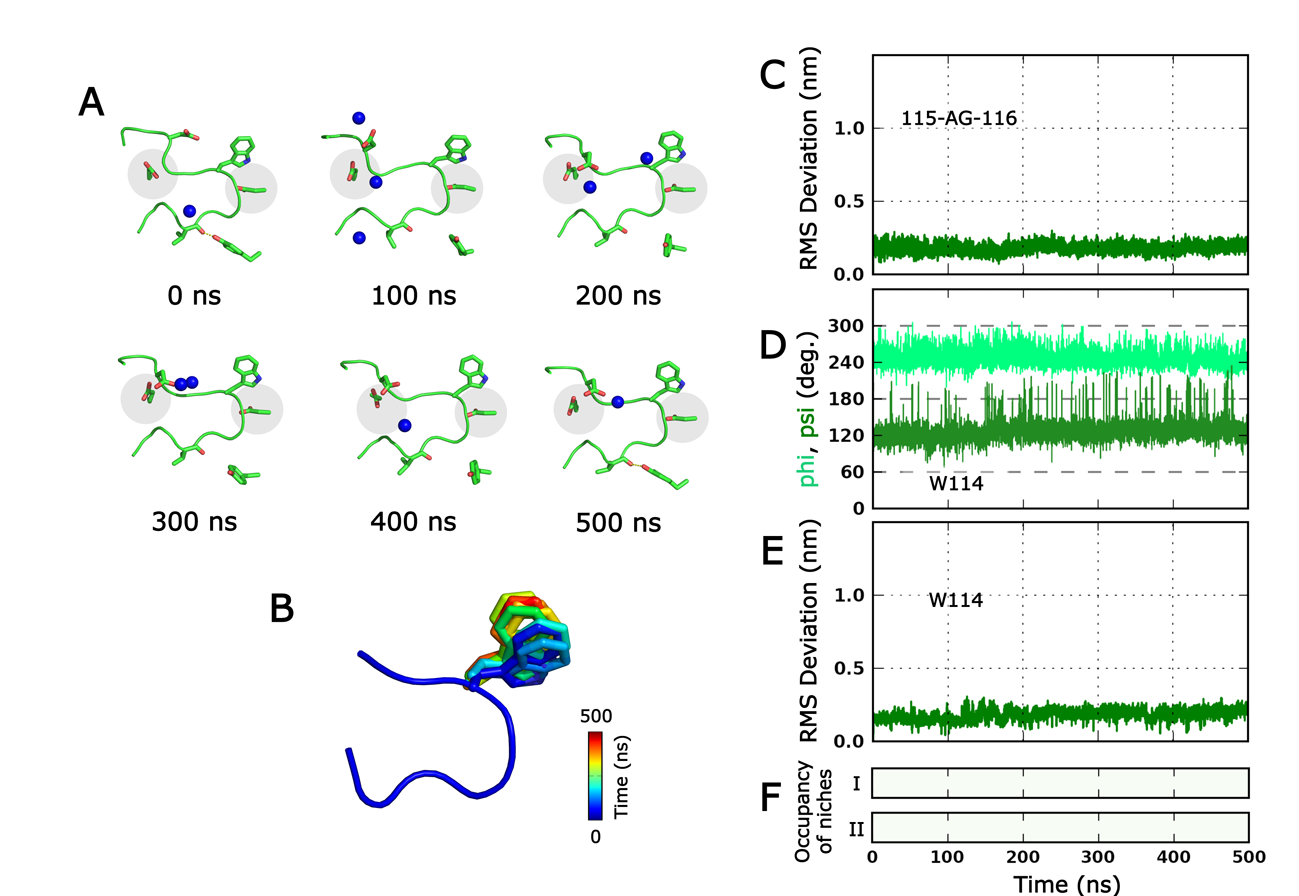

Supplement: S17 Fig — (A) Snapshots of L3 tip. In each snapshots, residues W107 to D120 are shown in cartoon; W114, D117, E266 and D321 in sticks; and K+ within 3.5 Å of the tip in blue spheres. The acidic niche I (E266) and niche II (D321) are highlighted in gray. (B) W111 movements taken every 50 ns. The RMSD of 115-AG-116 (C), the phi/psi angle of W114 (D) and the RMSD of W114 (E) are laid out to show the sequential events of exposure and binding of the two niches under L3 (F). (TIF) [file pcbi.1004255.s017.tif]

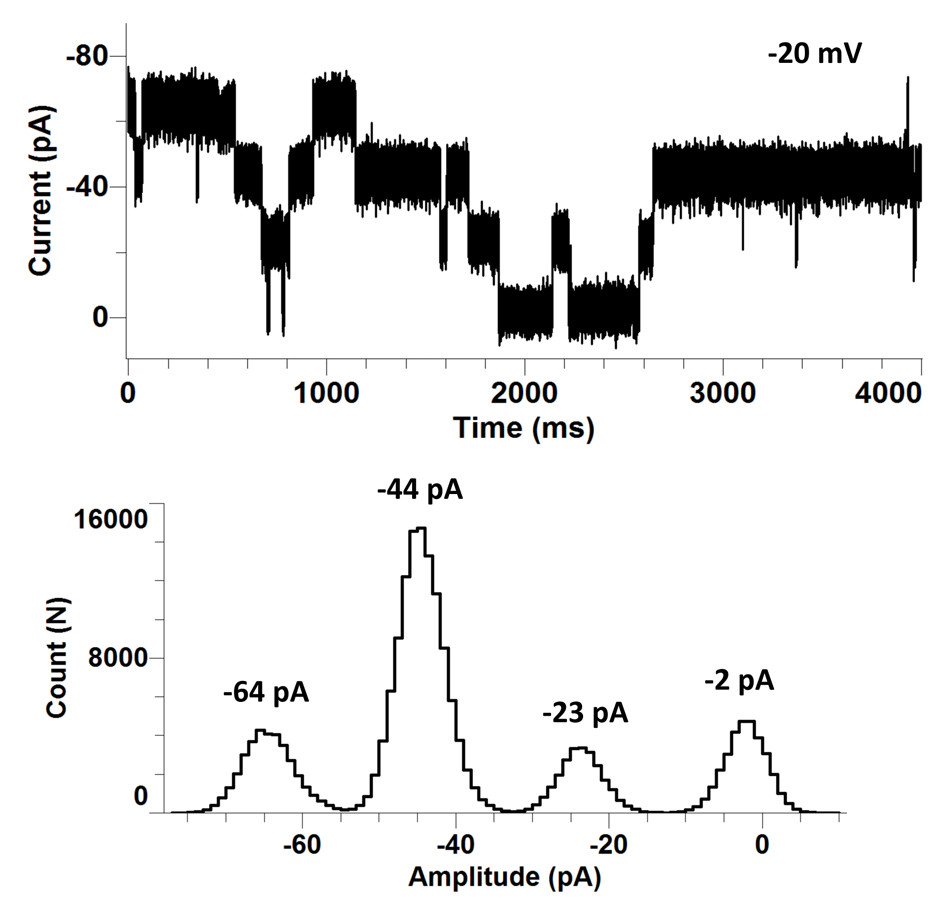

Supplement: S18 Fig — Upper panel: the ion current trace; Lower panel: the corresponding histograms of the current trace. Buffer conditions: 1M KCl, 10 mM HEPES at pH 7. (TIF) [file pcbi.1004255.s018.tif]

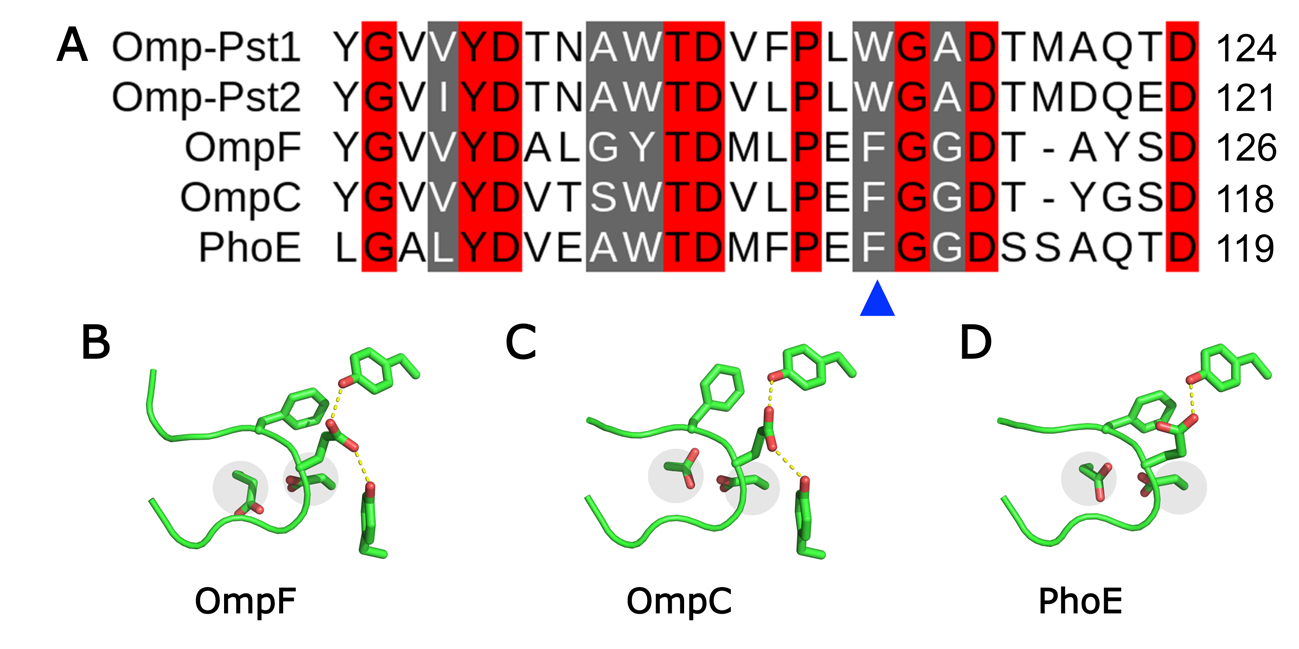

Supplement: S19 Fig — A) Sequence alignment of L3 of Omp-Pst1, Omp-Pst2, OmpF, OmpC and PhoE. Identical residues are highlighted in red and similar residues in gray. The residue that the blue triangle points out is the conserved aromatic residue at L3 tip. B) L3 tip of OmpF, OmpC and PhoE. The conserved aromatic residues are shown in sticks; the glutamic acid and the residue(s) it form hydrophone-bonding(s) with in sticks; and the main contributors of the acidic niches beneath L3 in sticks and highlighted in gray. (TIF) [file pcbi.1004255.s019.tif]
