# Supplementary material for: Understanding Voltage Gating of Providencia stuartii Porins at Atomic Level
Source: PLoS Comput Biol. 2015 May 8;11(5):e1004255. doi: 10.1371/journal.pcbi.1004255 (PMC4425685; doi:10.1371/journal.pcbi.1004255)
Supplement: S1 Table — (DOCX) [file pcbi.1004255.s020.docx]

**Table S1. System configurations:**

|  | **System Atoms** | **Lipids** | **Waters** | **Ions**  **(K-Cl)** | **Voltage**  **(V)** | **Simulation Length (ns)** |
| --- | --- | --- | --- | --- | --- | --- |
| Omp-Pst2 | 227,077 | 454 | 51,976 | 1,035-990 | -1 | 500 |
|  |  |  |  |  | +1 | 500 |
|  |  |  |  |  | 0 | 100 |
| Omp-Pst1 | 227,506 | 454 | 51,943 | 1,018-1,006 | -1 | 500 |
|  |  |  |  |  | +1 | 500 |
|  |  |  |  |  | 0 | 100 |
